# The Effect of Surface Composition on the Selective Capture of Atmospheric CO_2_ by ZIF Nanoparticles: The Case of ZIF-8

**DOI:** 10.1021/acs.jcim.2c00579

**Published:** 2022-09-23

**Authors:** Alexsander C. Vendite, Thereza A. Soares, Kaline Coutinho

**Affiliations:** † Instituto de Física, Universidade de São Paulo, Cidade Universitária, São Paulo 05508-090, Brazil; § Hylleraas Centre for Quantum Molecular Sciences, University of Oslo, 0315 Oslo, Norway

## Abstract

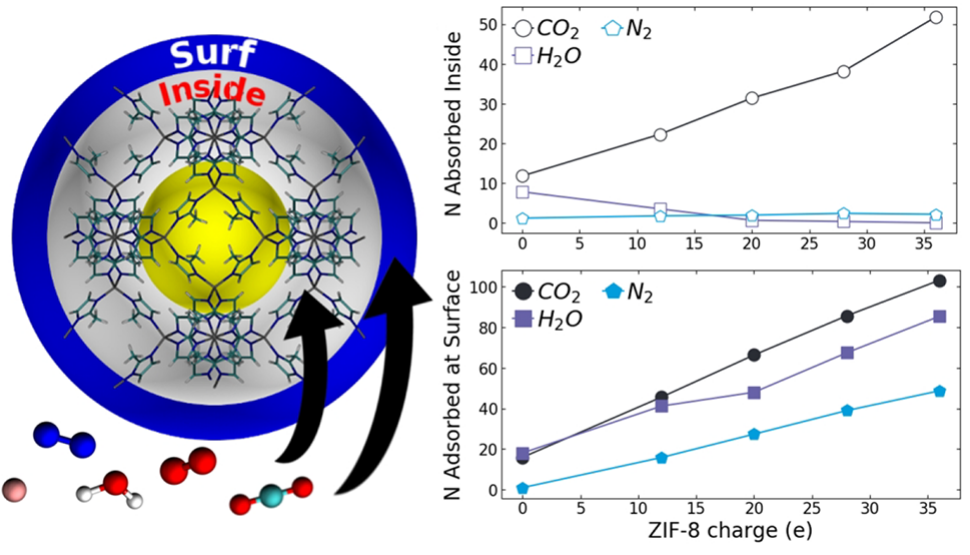

We performed theoretical studies of CO_2_ capture in atmospheric conditions by the zeolitic imidazolate framework-8 (ZIF-8) via classical Monte Carlo (MC) simulations with Metropolis sampling and classical molecular dynamics (MD) simulations in the NVT and NPT ensembles and different thermodynamic conditions. The ZIF-8 framework was described by varying unit cell dimensions in the presence of pure gases of CO_2_, N_2_, O_2_, Ar, and H_2_O steam as well as binary mixtures of CO_2_:N_2_ and CO_2_:H_2_O in s 1:1 concentration. Different chemical compositions of the framework surface was considered to provide an accurate treatment of charge and charge distribution in the nanoparticle. Hence, surface groups were represented as unsaturated zinc atom (Zn^+2^), 2-methylimidazole (mImH), and deprotonated 2-methylimidazole (mIm^–^). Force field reparameterization of the surface sites was required to reproduce the interactions of the gas molecules with the ZIF-8 surface consistent with quantum mechanics (QM) calculations and Born–Oppenheimer molecular dynamics (BOMD). It was observed that ZIF-8 selectively captures CO_2_ due to the negligible concentrations of N_2_, O_2_, Ar, and H_2_O. These molecules spontaneously migrate to the inner pores of the framework. At the surface, there is a competitive interaction between H_2_O, CO_2_, and N_2_, for the positively charged ZIF-8 nanoparticle with a large binding energy advantage for water molecules (on average −62, −15, and −8 kcal/mol respectively). For the neutral ZIF-8 nanoparticle, the water molecules dominate the interactions due to the occurrence of hydrogen bond with the imidazolate groups at the surface. Simulations of binary mixtures of CO_2_/water steam and CO_2_/N_2_ were performed to investigate binding competition between these molecules for the framework positively charged and neutral surfaces. It was found that water molecules drastically block the interaction between CO_2_ molecules and the framework surface, decreasing CO_2_ capture in the central pore, and CO_2_ molecules fully block the interaction between N_2_ molecules and the framework. These findings show that CO_2_ capture by ZIF-8 is possible in atmospheric environments only upon dehydration of the atmospheric gas. It further shows that ZIF-8 capture of CO_2_ from the atmospheric environment is dependent on thermodynamic conditions and can be increased by decreasing temperature and/or increasing pressure.

## 1 Introduction

Zeolitic imidazolate framework (ZIF) is an unique class of microporous materials with zeolite-like topologies of exceptional thermal, chemical, and mechanical stability.^1−3^ ZIFs are mostly made of tetrahedral units containing one divalent transition metal M^+2^ and four imidazolate anions.^1,2,4^ The strong coordination bond between the imidazolate linker and the metal center is pivotal for the exceptionally high thermal (>673 K) and moisture stabilities of this class of frameworks compared to most metal organic frameworks (MOFs). Furthermore, over 150 ZIF structures have been synthesized in a wide array of zeolite topologies (e.g., gmelinite, sodalite, rho) and a broad repertoire of chemically modified imidazolate linkers. The easy preparation; topological control; high porosity; and chemical, mechanical, and thermal stability of ZIF structures has enabled their use for numerous applications, which have been explored in several recent reviews.^5,6^ One of the most promising applications of ZIFs is the sequestration, capture, and separation of gases, particularly CO_2_.^3,7,8^ Among the zeolitic imidazolate frameworks, ZIF-8 is regarded as a singular candidate for CO_2_ capture.^9−11^ It has been one of the most extensively studied ZIFs because of its highly effective aperture size (∼3.4 Å) for the molecular selectivity of many gases of interest (e.g. H_2_, CO_2_, O_2_, N_2_, CH_4_).^12^ For instance, ZIF-8 nanoparticles adsorbs ∼14 and ∼8 times more CO_2_ than CH_4_ or N_2_ in binary mixtures.^13,14^ It displays gas saturation capacities of 55.32 ± 0.01 (CO), 55.00 ± 0.02 (N_2_), 64.83 ± 0.04 (O_2_), and 61.37 ± 0.02 (Ar) molecules per unit at 77 K.^15^ Chemical modifications of ZIF-8 can increase CO_2_ adsorption capacities 1 bar and 273 from 38.3 to 97.6 cm3g^–1^ or over ca. 118%.^16,17^ Likewise, the selectivity of chemically modified ZIF-8 for CO_2_/N_2_ at 0.01 bar and 298 K can be increased by up to 173.0% compared to the unmodified ZIF-8.^17^

ZIF-8 exhibits high CO_2_ uptake capacity (e.g., 9.1 mol kg^–1^ at 303 K and 45 bar)^18^ and outstanding stability in rather distinct environments such as refluxing organic solvents, water, atmospheric air, aqueous alkaline solution and water-dissolved SO_2_ gas.^1−3^ ZIF-8 is comparatively flexible with respect to most zeolites. Gas permeation experiments have shown that molecules with a diameter larger than 3.4 Å can slowly diffuse through the framework.^19^ Such structural flexibility has been associated with the rotation of imidazole ring around the N–N axis (gate-opening) and the bending of the methyl group away from the plane defined by the imidazole ring (pore-breathing).^20,21^ The subtle dynamics of the organic linker leads to an increase in the ZIF-8 pore aperture of 0.7 Å.^22^ Recent X-ray absorption spectroscopy measurements estimate that the gate opening transition lies between 50 and 100 mbar at 80 K,^21^ whereas previous reports show that the gate-opening transition takes place at high CO_2_ loadings.^23−25^

Previous computational simulations have provided useful insights on CO_2_ selectivity and capture by ZIF-8.^1,11,23,26−42^ It has been shown that CO_2_ adsorbs to bulky ZIF-8 at several sites within the framework pores: near the imidazolate rings and above the three methyl rings,^30,31^ in the cage center,^32^ near the H atoms^26^ and on both sides of the six ring windows, above the four ring windows.^28^ CO_2_ adsorption increases with the pressure,^27,29,31−33,36^ and decreases with the increase of temperature.^29^ Likewise, the adsorption of N_2_, O_2_, H_2_O, CO_2_/N_2_, and CO_2_/CH_4_ gas mixtures to ZIF-8 also has been shown to increase with pressure,^27,29,36,37^ though H_2_O adsorption to bulky ZIF-8 occurs exclusively at high pressures.^34^ Computation simulations have shown that ZIF-8 is selective for CO_2_ over N_2_^35^ with no significant effect due to the presence of other gases such as O_2_, H_2_O, or SO_2_.^27^ Higher CO_2_/N_2_ separation was observed at lower temperatures.^30^ Furthermore, CO_2_/CH_4_, CO_2_/N_2_, and CO_2_/H_2_ selectivity toward CO_2_ has been shown to grow with increasing pressure.^32,33,36,37^ Moreover, the CO_2_/CH_4_ selectivity is dependent on the H_2_O ratio and temperature.^1^ Computational simulations have also suggested that, in the presence of CO_2_ and CO_2_/N_2_ at high pressures, a swing effect in the imidazolate linkers allow for the gate opening of the smaller pore windows in the bulky ZIF-8 structure.^30,38^ Although the structural flexibility of ZIF-8 has been shown to have an insignificant effect on gas adsorption, it appears to be important for the diffusion,^32,40,41^ particularly for molecules larger than the pore window.^42^ The diffusivities of CO_2_ in pristine and CO_2_/CH_4_ mixtures have been shown to barely change with pressure, and CO_2_ diffuses at similar rates in both pristine CO_2_ and the binary mixture.^32^ The mean residence time of CO_2_ molecules inside bulky ZIF-8 increases at higher pressures and lower temperatures.^28^ These simulations made use of computational methods such as Grand canonical Monte Carlo (GCMC), molecular dynamics (MD) and GCMC/MD hybrid coupled to periodic boundary conditions and temperatures near to 273 K. A variation of this theme was performed by Wu et al., who applied GCMC to simulate N_2_ adsorption to a partially flexible ZIF-8 nanoparticle at 77 K.^11^ Less commonly, Born–Oppenheimer MD (BOMD) simulations have also been performed to develop force field parameters for classical MD.^43^ There is one work that deviated from the pattern with a partially flexible GCMC of ZIF-8 represented by ca. 3000 atoms nanoparticle and a periodic bulk.^39^ The neutral nanoparticle was constructed as a sphere of 5 nm of diameter and undercoordinated Zn atoms terminated with imidazole or hydroxyl groups and exposed N atoms protonated. The simulations have shown that the structural transition of ZIF-8 happens at a higher pressure in the bulk compared to the nanoparticle, and in the latter, it is independent of the tested surface compositions. It has further been shown that the pressure needed for the structural transition depends on the particle size.

These computational efforts have focused on the representation of the inner pore surface (i.e., the bulk structure), and with a few exceptions,^39^ the influence of the particle shape and surface composition on gas adsorption is neglected. On the other hand, recent experimental studies have attested to the relevance of particle size and surface composition for the structural stability, adsorbate upload and diffusion rates, and surface reactivity.^1,36,44−46^ Although bulk ZIF-8 has been shown to be stable under dilute solutions of aqueous SO_2_, the two predominant crystallographic facets of the framework exhibit different degradation and bulky adsorbate diffusion rates. The latter differs in ca. 1 order of magnitude between the two types of facets.^47^ Recent reports have also disputed the chemical and structural resistance of ZIF-8 to water and to long-term CO_2_ exposure in water under ambient conditions.^44−46,48,49^ Accordingly, the hydrolysis of ZIF-8 is presumed to occur through the partial cleavage of Zn–N bonds at the framework surface with release of Zn^+2^ and imidazolate ligands in the aqueous environment.^46,49^ The dissolution process is halted when the concentration of the reactants in the liquid phase achieves equilibrium or upon addition of 2-methylimidazole to the solution. It should be noticed that the dissolution of ZIF-8 in water increases with decreasing ZIF-8/water weight ratio, regardless of ZIF-8 crystallite sizes and preparation methods.^46^ As for ZIF-8 in the presence of CO_2_ and water for long enough time,^45,48^ a putative two step reaction takes place to yield zinc carbonate (or zinc carbonate hydroxides) and 2-methylimidazole.^48^ This reaction can be prevented by previous dehydration and/or deacidification of the fluids to be treated. Remarkably, CO_2_ capture by ZIF-8 is substantially increased under the existence of liquid water.^48^

The accurate description of the framework surface chemical composition is a precondition to fully understand gas selectivity and capture by ZIF-8 under varying conditions. ZIF-8 has a composition of 42.0 for C, 4.5 for H, and 24.0 for N in weight %. Although these values can exhibit small deviations depending on the experimental conditions of synthesis, the Zn ratios exhibit fairly larger deviations within the range of 22–30 wt %.^26−29,33^ Interestingly, there is also evidence of the presence of O atoms in the ZIF-8 structure, albeit in small proportions.^27−29,33,36^ Hence, this data indicates that ZIF-8 structures exhibit differences in the ratio of surface metal sites accordingly to the synthesis protocol and environmental conditions. Moreover, Zn atoms on the ZIF-8 surface will not be tetracoordinated as in the inner region, but will instead be coordinated to 2 or 3 imidazolate ligands.^28,29,37,50^ This has been demonstrated by an elegant experiment where ZIF-8 nanoparticles were assembled in different crystalline morphologies: cubic with 6 (100) faces, rhombic dodecahedral with 12 (110) faces, or truncated rhombic dodecahedral with 6 (100) and 12 (110) faces.^29^ These nanoparticles were subsequently coated with an Au layer via a surfactant-mediated process. The surfactant could only bind to Zn atoms coordinated to 2 imidazole ligands, which were most of the surface metals. Therefore, it is plausible to assume that variations of Zn weight percentage in the ZIF-8 structure may be due to differences in the nanoparticle surface-to-volume ratio and/or the synthesis-dependent surface composition. Furthermore, it has been previously shown that strong Lewis and Brønsted acid/basic sites exist at the external surface or at defects, but not in the microporosity of ZIF-8.^27^ These surface sites correspond to a large assortment of chemical groups such as OH and NH groups, hydrogen carbonates, low-coordinated Zn atoms, and free N^–^ moieties from surface imidazolates that easily react with water and its dissociation products OH^–^ and H^+^ in aqueous solvent.^28,29,36,37^ The ratio of the surface chemical sites is governed by operating conditions (temperature and pressure) and is not easily detectable through X-ray crystallography measurements.^1,38^

**Figure 1:**
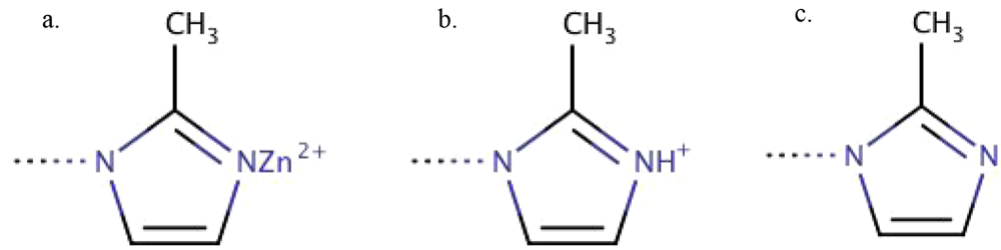
Chemical structures of surface sites (X_surf_) on the ZIF-8 nanoparticle described by [(Zn^+2^)*_l_* (mIm^–^)_*m*_ (X_surf_)*_n_*]: (a) X_surf_ = Zn^+2^ atom monocoordinated with one imidazole group, (b) X_surf_ = H^+^ atom and (c) none. For the 1 × 1 × 1 nanoparticle *l* = 24, *m* = 60, and *n* = 24, for the 2 × 2 × 2 nanoparticle *l* = 144, *m* = 336, and *n* = 72 and for the 3 × 3 × 3 nanoparticle *l* = 432, *m* = 972, and *n* = 144.

**Figure 2:**
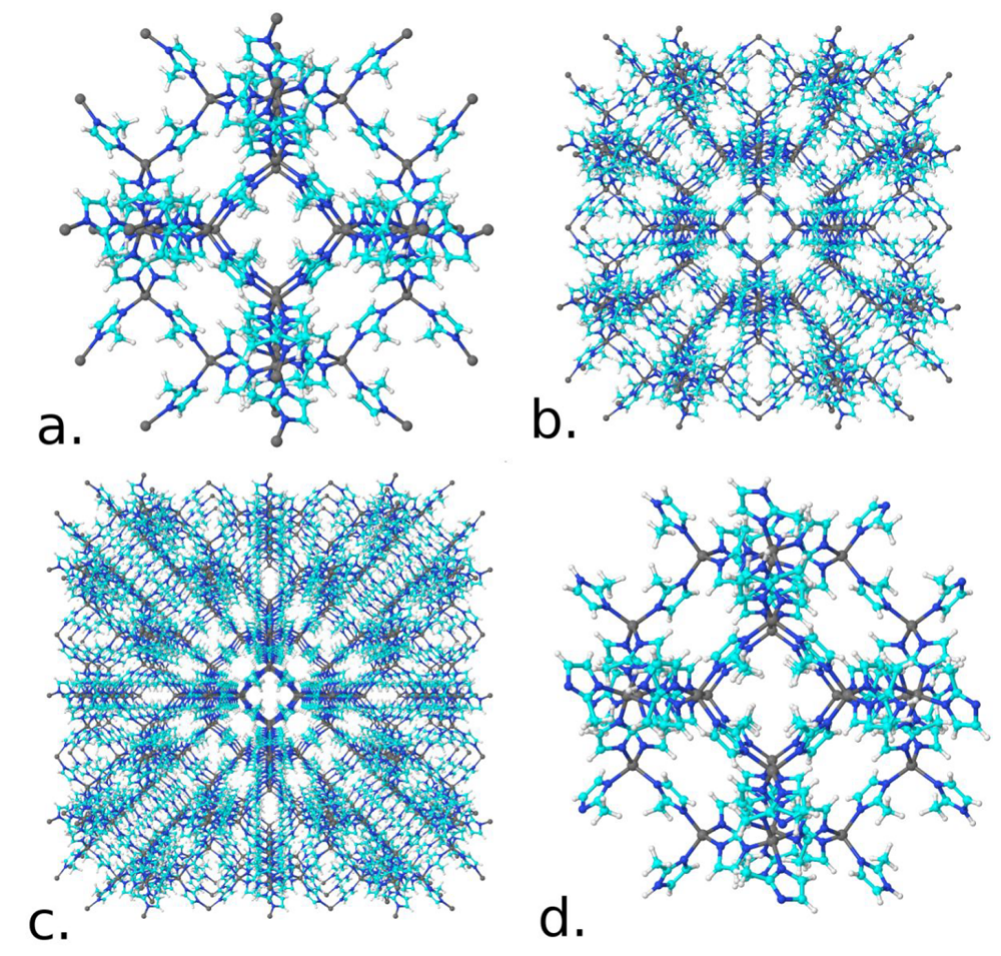
Molecular representation of ZIF-8 nanoparticles with different packing size: (a) 1 × 1 × 1 with 708 atoms and diameter around 3 nm, (b) 2 × 2 × 2 with 3912 atoms and diameter around 5.5 nm, and (c) 3 × 3 × 3 with 11268 atoms and diameter around 8.5 nm, all three nanoparticles with all surface sites with Zn^+2^ atoms, and (d) 1 × 1 × 1 with 12 surface sites with H^+^ and 12 with bare nitrogen atoms. These structures were generated with software Mercury^51^ using the ZIF-8 unit cell were taken from the crystallographic structure^1^ and the 1 × 1 × 1, 2 × 2 × 2 and 3 × 3 × 3 packing with sodalite (SOD) topology. The color representations of the atoms are cyan (carbon), blue (nitrogen), white (hydrogen), and gray (zinc).

In this work, we address the role of the surface composition of ZIF-8 nanoparticles on the spontaneous diffusion of atmospheric gases (CO_2_, H_2_O, N_2_, O_2_, and Ar) via classical Monte Carlo (MC) and classical molecular dynamics (MD) simulations in the NVT and NPT ensembles and varying conditions of temperature and pressure. For the case of the 1 × 1 × 1 nanoparticle, a core region is composed by 24 Zn^+2^ tetracoordinated atoms (Zn^+2^)_24_ and 60 2-methylimidazole groups (mIm^–^)_60_ and a surface region is composed by 24 surface sites (X_surf_)_24_, i.e., [(Zn^+2^)_24_(mIm^–^)_60_(X_surf_)_24_]. Three chemical representations of the X_surf_ were considered: Zn^+2^, H^+^, and the bare nitrogen atom without binding to the latter atoms (Figure 1). Therefore, the total charge of the 1 × 1 × 1 ZIF-8 nanoparticle depends on the surface composition and was allowed to vary from +36*e* to zero. This range was chosen because it has been shown that the surface of ZIF-8 has a positive Zeta potential.^27^ Furthermore, the choice of the smallest crystal unit representative of ZIF-8 makes surface defects fully negligible, providing a truly microscopic benchmark against which macroscopic experimental measurements accounting for surface defects can be compared. Larger ZIF-8 nanoparticles, 2 × 2 × 2 and 3 × 3 × 3, were considered as well to evaluate the trend for size increments. Their representations were composed as [(Zn^+2^)_144_(mIm^–^)_336_(X_surf_)_72_] and [(Zn^+2^)_432_(mIm^–^)_972_(X_surf_)_144_], respectively, with X_surf_ = Zn^+2^. The atomistic representations of the three simulated ZIF-8 nanoparticles are shown in Figure 2 were all surface sites X_surf_ have Zn^+2^ atoms. Depending on the packing size of the nanoparticles, it is possible to see surface Zn^+2^ atoms coordinated with one (Figure 2a) and two imidazolate linkers (Figures 2b and 2c). The surface sites X_surf_ of the 1 × 1 × 1 nanoparticle were modified with the addition of 12 sites with H^+1^ atoms and 12 sites with bare nitrogen atoms randomly distributed (Figure 2d).

## 2 COMPUTATIONAL METHODS

A refined set of atomic charges was assigned to each one of different chemical groups on the ZIF-8 surface and unavailable in the original set of atomic parameters.^52^ Therefore, for the 1 × 1 × 1 ZIF-8 nanoparticle, there was a core region composed by 24 bulk zinc atoms (i.e., saturated or tetracoordinated, Zn^+2^) and 60 deprotonated 2-methylimidazole groups (mIm^–^), and a surface region composed by 24 surface sites (X_surf_) considered to be unsaturated zinc atoms (X_surf_ = Zn^+2^), protons (X_surf_ = H^+^) or bare N atoms of the methylimidazole group (Figure 1 and 2). The charge of the core region, [(Zn^+2^)_24_(mIm^–^)_60_] is −12*e*, whereas the charge of the surface region depends on its composition. Eight nanoparticles [(Zn^+2^)_24_ (mIm^–^)_60_ (X_surf_)*_n_*] with different surface compositions were simulated by using MC method: two neutral nanoparticles, *Q* = 0, containing at the surface either 6 randomly distributed unsaturated zinc atom, [(Zn^+2^)_24_(mIm^–^)_60_(Zn^+2^)_6_], or 12 protons, [(Zn^+2^)_24_(mIm^–^)_60_(H^+^)_12_]; and six positively charged nanoparticles presenting at the surface *n* = 12, 16, 20, and 24 Zn^+2^ with total charge of *Q* = +12*e*, +20*e*, +28*e*, and +36*e*, respectively, and *n* = 18 and 24 H^+^ with *Q* = +6*e* and +12*e*, respectively. The system [Zn^+2^mIm^–^] was also simulated via MC to allow for comparisons between the classical simulations and QM calculations. In a similar fashion, the 2 × 2 × 2 and 3 × 3 × 3 ZIF-8 nanoparticles were constructed as [(Zn^+2^)_144_(mIm^–^)_336_(X_surf_)_72_] and [(Zn^+2^)_432_(mIm^–^)_972_(X_surf_)_144_], respectively, with X_surf_ = Zn^+2^. All simulated systems are listed in the Table 1 and in the Tables S3–S5 in the Supporting Information (SI) file.

**Table 1:** Simulated Systems. Monte Carlo (MC) and Molecular Dynamics (MD) Simulations Were Performed for the ZIF-8 Nanoparticle and Its Simplest Constituting Chemical Unit in the Presence of Atmospheric Gases at Different Ensembles, Temperature, and Pressure Conditions^a^

method	solute	(X_surf_)_*n*_	gas	*T* (K)	*P* (atm)
MC	[(Zn^+2^)_24_(mIm^–^)_60_(X_surf_)_*n*_]	(Zn^+2^)_24_	CO_2_, H_2_O, N_2_, O_2_, Ar, CO_2_:H_2_O, CO_2_:N_2_	273	1
		(Zn^+2^)_*n*_^b^	CO_2_, H_2_O, N_2_	273	1
		(H^+^)_*n*_^c^	CO_2_, H_2_O, N_2_	273	1
		(Zn^+2^)_24_	CO_2_, H_2_O	298	1
		(Zn^+2^)_6_	CO_2_	298	1
		(Zn^+2^)_24_	CO_2_	273	1, 20
	[Zn^+2^mIm^–^]	−	CO_2_, H_2_O, N_2_	273	1
			H_2_O	298	1, liq
MD	[(Zn^+2^)_24_(mIm^–^)_60_(Zn^+2^)_24_]	−	CO_2_	273	1
	[(Zn^+2^)_144_(mIm^–^)_336_(Zn^+2^)_72_]	−	CO_2_	273	1
	[(Zn^+2^)_432_(mIm^–^)_972_(Zn^+2^)_144_]	−	CO_2_	273	1

a See more details in Tables S2 and S3.

b *n* = 6, 12, 16, 20.

c *n* = 12, 18, 24.

### 2.1 Monte Carlo Simulations of Gas Adsorption to ZIF-8 Nanoparticles

We have performed classical Monte Carlo (MC) simulations with the Metropolis sampling algorithm in NVT and NPT ensembles. The simulated systems were composed by one ZIF-8 nanoparticle and *N*_gas_ gas molecules, in total *N* = 1 + *N*_gas_ molecules with *N*_gas_ varying from 100 and 2000. For a given thermodynamic condition and ensemble, the ideal gas equation was used to calculate the initial cubic box length. For example, in the NVT ensemble for *T* = 273 K, *P* = 1 atm and *N*_gas_ = 100, 200, 500, 1000, and 2000, the cubic box lengths are 155.0, 195.3, 265.1, 333.9, and 420.7 nm, respectively. MC simulations were performed for the rigid ZIF-8 nanoparticle in the presence of single gas types (N_2_, O_2_, Ar, H_2_O and CO_2_) and binary mixtures of CO_2_:N_2_ and CO_2_:H_2_O in 1:1 at a concentration of 1:1. Because the atmospheric gas composition is severely unbalanced for some molecules, such as CO_2_ with concentration around 360–400 ppm, we performed MC simulations of the ZIF-8 nanoparticle with a single gas type (N_2_, O_2_, Ar, H_2_O and CO_2_) as well as binary mixtures, to avoid poor sampling for the gases occurring in very small amounts in the atmosphere. In the NVT ensemble, the density of the ideal gas was kept constant, while in the NPT, the pressure was kept constant, and the density was free to fluctuate. Simulations were performed at two temperatures, 273 and 298 K, since both temperatures are common in the atmospheric environment. Periodic boundary conditions were applied to a cubic box with the image method using a cutoff radius of 6 nm larger than the nanoparticle radius; that is, for a 1 × 1 × 1 nanoparticle of radius 1.5 nm, the cutoff radius will be 7.5 nm. In liquid-phase simulations, the computational cost of computing interactions between all atoms of numerous solvent molecules requires the use of long-range corrections like the different flavors of the Particle Mesh Ewald approach. Therefore, nonbonded interactions for atoms within the cutoff radius are treated explicitly, whereas nonbonded interactions for atoms beyond the cutoff radius are approximated via long-range corrections. Since these corrections require a neutral simulation box and the simulated ZIF-8 nanoparticles may have a total charge (*Q* varying from +36*e* to 0), it was opted to treat all nonbonded long-range interactions explicitly in the simulated systems to avoid the addition of more charged particles in the form of counterions. The use of the outsized cutoff radius of 6 nm ensures that all important interactions between the molecules were considered. The chosen cutoff value was determined from analysis of the radial distribution functions (RDF) that displayed values equal to 1 for distances much smaller than 6 nm beyond the nanoparticles, and hence exhibiting an ideal gas, or noninteracting, system behavior. Most importantly, the inclusion of counterions in the system to compensate for the ZIF-8 nanoparticle total charge and satisfy the system neutrality of long-range corrections can artificially change the distribution of gas molecules in the simulation due to Coulombic interactions between counterions and the atomic charges of the gas molecules, as well as generate counterion distributions near the nanoparticles that can block the interaction with the gas molecules. Therefore, the usage of oversized cutoff radius and the explicit treatment of long-range interactions instead of long-range corrections bypass the need of addition of numerous counterions to neutralize the simulation box though at increased computational cost.

**Figure 3:**
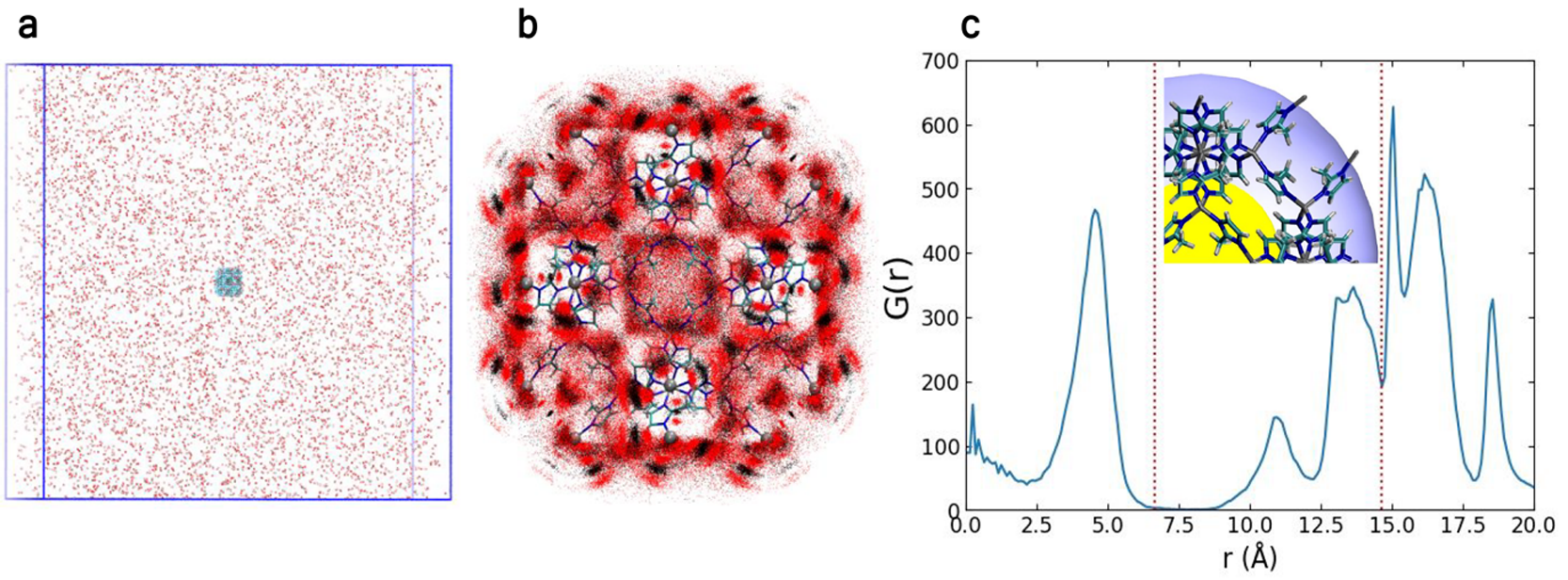
Illustration of the (a) initial configuration of the MC simulation in the NVT ensemble of one ZIF-8 nanoparticle of 1 × 1 × 1 packing with all 24 surface sites with Zn^+2^ atoms (708 atoms and radius around 1.5 nm) in CO_2_ gas with 1000 molecules at temperature of 273 K, pressure of 1 atm and cubic box length of 333.9 nm. (b) Close-up of the final configuration showing the CO_2_ molecules inside and at the surface of the nanoparticle. (c) Radial distribution function between the center-of-mass of the ZIF-8 and CO_2_ molecules. The two vertical lines define the radius of the central pore at 6.5 Å (yellow region shown in the inset image) and the radius of the inside region at 14.7 Å (light blue shown in the inset image). The color representations of the atoms are cyan (carbon), blue (nitrogen), red (oxygen), white (white), and gray (zinc).

The simulated molecules were allowed to translate and rotate accordingly to the intermolecular interactions modeled by the standard Coulomb and Lennard-Jones potentials with the Lorentz–Berthelot combination rules for the atomic parameters. The *q*_*i*_, ε_*i*_, and σ_*i*_ atomic parameters were taken from AMBER force field previously adapted for ZIF-8 by Hu et al.^52^ Since the latter contained only atomic parameters for the bulky imidazolate and Zn^2+^ ions of ZIF-8, it was necessary to calculate new sets of atomic charges for the remaining representations of surface chemical sites via QM calculations as described in the next section. The AMBER force field was used in the atomistic description of the ZIF-8 nanoparticle with nonbonded parameters described in Table 2 and bonded parameters in Table SI–S1. The force field parameters from Vujić et al.^53^ were used to describe gas molecules CO_2_, N_2_, O_2_, and Ar combined with the SPC/E model for the water steam.^54^ The initial configurations were generated with the nanoparticle at the center of the simulation box and gas molecules were added at random positions and orientations. A total of 2.0 × 10^6^ and 1.0 × 10^6^ MC cycles were performed during the equilibration and production phases of the simulations, respectively, where in 1 MC cycle, *N* molecules were randomly selected and attempted to move. Representations of the initial and final configurations from the NVT simulation of 1 × 1 × 1 ZIF-8 nanoparticle with 1000 CO_2_ gas molecules at temperature of 273 K, pressure of 1 atm and cubic box length of 333.9 nm is shown in Figure 3. In the production phase, configurations of the simulated system were saved at every 100 cycles and analyzed with respect to radial distribution functions, the number of gas molecules captured inside and on the surface of the nanoparticle, binding energies, and minimum distances between ZIF-8 and the gas molecules. All MC simulations and analyses were performed with the DICE software.^55^

**Table 2:** Force Field Atomic Parameters (*q*_*i*_, ε_*i*_, and σ_*i*_) for the Nonbonded Coulomb and Lennard-Jones Potentials Used in the MC Simulations of the ZIF-8 Nanoparticles^a^

bulk atoms^b^	*q*_*i*_ (*e*)	ε_*i*_ (kcal/mol)	σ_*i*_ (Å)
Zn	1.000	0.0125	1.960
N	–0.500	0.1700	3.250
C1	0.500	0.0860	3.400
C2	–0.100	0.0860	3.400
HC2	0.100	0.0150	2.421
C3	–0.300	0.1094	3.400
HC3	0.100	0.0157	2.650
surface sites^c^			
(Zn)_*n*_	1.000 (*n* = 6); 1.500 (*n* = 12); 1.625 (*n* = 16); 1.700 (*n* = 20); 1.750 (*n* = 24)	0.0125	1.960
(N)_*n*_	–0.320 (*n* = 12); −0.183 (*n* = 18); −0.125 (*n* = 24)	0.1700	3.250
(HN)_*n*_	0.320 (*n* = 12); 0.350 (*n* = 18); 0.375 (*n* = 24)	0.0157	1.069

a The bonded parameters are described in Table S1.

b from Hu et al.;^52^

c Proposed in this work based in QM cluster calculations (section 2.3).

### 2.2 Molecular Dynamics Simulations of Gas Adsorption to ZIF-8 Nanoparticles

Classical molecular dynamics simulations were conducted with the leapfrog algorithm in NVT ensemble using the velocity-rescale thermostat. The 1 × 1 × 1, 2 × 2 × 2 and 3 × 3 × 3 ZIF-8 nanoparticles were simulated as flexible frameworks with 1000, 8000, and 27 000 CO_2_ molecules, respectively. Alike to the MC simulations, temperature of 273 K, pressure of 1 atm, and the same initial configurations, periodic boundary conditions, cutoff radius and nonbonded force field parameters were used. An exception to the latter are the charges of the surface Zn atoms with 1.70 and 1.65*e* on the monocoordinated Zn atom for 2 × 2 × 2 and 3 × 3 × 3, respectively, and 1.60*e* on the dicoordinated Zn atom for both structures. The systems were equilibrated for 10 ns and production phase lasted 90 ns. The H bonds were constrained by the LINCS^56^ algorithm with a time step of 2 fs. The bonded force field parameters for CO_2_ were *k*_b_^CO^ = 714230.5754 kJ/(mol nm^2^) and *k*_θ_^OCO^ = 1236.1252 kJ/(mol rad^2^) based on the EPM2^57^ and UFF^58^ models. All MD simulations were performed with the GROMACS software v.5.1.4.^59,60^

The fully flexible MD simulation of the 1 × 1 × 1 nanoparticle in pristine CO_2_ gas was also used to evaluate whether the approximation of the nanoparticle as a rigid framework was reasonable. The time-dependent root-mean-square deviation (RMSD) between the heavy atoms of the simulated system with respect to the ZIF-8 crystallographic structure remained approximately constant at 1 Å throughout the simulated time (Figure S6). The discrete RMSD difference can be assigned to the slight displacement of the unsaturated Zn atoms at the surface, the free rotation of the methyl groups and small rotation of the imidazole groups around the N–N axis. The organic linker flexibility has been described as well in previous works.^20−22^ Although it represents a small change in the overall structure of the ZIF-8 causing variations around 1 Å in the RMSD, it triggers a slight opening of the pores and a meager enhancement of gas capture. For the CO_2_ case, there is an increase of 19% in the inner region capture and 14% at the surface. Hence, the treatment of the ZIF-8 nanoparticle as a rigid system for the purpose of investigating the role of surface composition on the interactions with atmospheric gases is an acceptable approximation.

### 2.3 Quantum Mechanics Calculations

Geometry optimization, atomic charge, and binding energy calculations were performed using the density functional theory with the B3LYP exchange-correlation functional^61^ and the Dunning basis set with correlation consistent double-ζ basis functions augmented with a set of diffuse functions (so-called aug-cc-pVDZ).^62^ The ZIF-8 initial geometry used in the calculations was taken from the crystallographic structure.^1^ Atomic charges for the surface sites *X*_surf_ were calculated with the CHELPG procedure^63^ that is based on estimates of the solvent accessible surface and a grid to identify the best set of atomic charges to fit the QM electrostatic potential of the system. The solvent accessible surface was generated using default values for the van der Waals radius of the atoms as implemented in Gaussian09 software^64^ and 0.95 Å for the Zn atom. One set of calculations was performed for atomic coordinates composed of one saturated zinc atom coordinated with two mIm^–^ and two mImH groups [Zn^+2^(mIm^–^)_4_(H^+^)_2_]. For this first system, we tested the Hartree–Fock method (HF) and several density functionals (GGA and hybrid, with and without dispersion, and with and without long-range correction), different basis sets (Pople series and correlation consistent), methods to calculate atomic charges (CHELPG, Merz–Singh–Kollman, and Hirshfeld), and values of the van der Waals radius of the Zn atom (from 0.7 to 2.6 Å) to identify the combination of QM options that best describes the atomic charges of the bulk ZIF-8 force field previously adapted by Hu et al.^52^ The best combination in the QM options was B3LYP/aug-cc-pVDZ/CHELPG/r(Zn) = 0.95 Å given priority to the atomic charges of Zn and N atoms. Using the same QM options, we generated other cluster systems to analyze the surface effect on the atomic charges concerning the unsaturation Zn with mono- and dicoordination with imidazole groups (Figure S2), and the polarization due to the interaction with gas molecules, selecting X_gas_ = CO_2_ and H_2_O, as nonpolar and polar molecules, respectively. Then, we optimized the geometries and calculated the atomic charges for the systems: monocoordinated [Zn^+2^mIm^–^] without gas molecules and [Zn^+2^mIm^–^]+(X_gas_)_3_ with three gas molecules, and dicoordinated [Zn^+2^(mIm^–^)_2_] without gas molecules and [Zn^+2^(mIm^–^)_2_]+(X_gas_)_2_ with two gas molecules.

To validate the atomic charges of the nanoparticle surface atoms, we compared the binding energies calculated with the classical force field for different systems, such as the gas molecules with the 1 × 1 × 1 nanoparticle [(Zn^+2^)_24_(mIm^–^)_60_(Zn^+2^)_24_] and with small simulated systems or optimized clusters, [Zn^+2^mIm^–^] and [mImH], with different quantities of gas molecules. Further, the classical and quantum-calculated binding energies were compared for small simulated systems or optimized clusters. The QM calculated binding energies were corrected with the base set superposition errors (BSSE) using the Counter Poise correction^65^ with B3LYP/cc-pVDZ under the geometry optimized with B3LYP/aug-cc-pVDZ. The optimized clusters are [Zn^+2^mIm^–^]+(H_2_O)_3_; [Zn^+2^mIm^–^]+(CO_2_)_*n*_ with *n* = 3, 4 and 5; and [Zn^+2^mIm^–^]+(N_2_)_*n*_ with *n* = 2 and 3. The values of *n* were selected based on the average number of gas molecules in the vicinity of the Zn^+2^ atoms as sampled with MC simulations. We select the same functional used in the geometry optimization because the idea is to validate the classical force field, comparing the order of magnitude of the binding energy calculated with classical and quantum methods, but not obtain the most precise values with QM calculations. Additionally, all the optimized clusters are positively charges (*Q* = +1); therefore, the most important contribution to the binding energy should be the Coulombic interaction and the dispersion may have a small contribution. All the QM calculations were performed with the Gaussian09 software.^64^

### 2.4 *Ab Initio* Molecular Dynamics

Born–Oppenheimer molecular dynamics (BOMD) simulations were performed for systems [Zn^+2^mIm^–^]+(X_gas_)_4_, where X_gas_ = CO_2_ or H_2_O in order to evaluate the reliability of the employed classical force field to describe the interactions between the Zn^+2^ atom and gas molecules. The BOMD simulations were performed in the NVT ensemble in a cubic box with size of 20 Å. The canonical sampling through velocity rescaling (CSVR) thermostat^66^ was used to maintain the temperature at 298 K. Both simulations were carried out with the Perdew–Burke–Erzernhof (PBE) exchange correlation functional,^67^ the hybrid Gaussian and plane-wave functions (GPW)^68^ and core electrons, Goedecker, Teter and Hutter (GTH) norm-conserving pseudopotential^69^ through the CP2K/Quickstep code.^70^ The Gaussian orbitals were depicted by a double-ζ-valence-polarization (DZVP) and the plane wave had a charge density cutoff of 280 Ry. The electronic density calculations had a self-consistent-field energy threshold of 10^–6^ hartree. Simulations were run for 40 ps with a time step of 0.25 fs.

## 3 Results and Discussion

### 3.1 Assessment of Multiple Representations of Surface Site Charges

The variable coordination number of Zn atoms on the ZIF-8 surface required different atomic charges from the representation of the system as containing exclusively bulk Zn atoms (Figures 1 and 2). This was necessary since the total charge of the whole nanoparticle, obtained by adding the charges of mIm^–^, Zn^+2^, and H^+^, was not attained with the bulk force field parameters applied to all atoms. Therefore, QM calculations were performed to obtain atomic charges for each one of the possible representations of chemical sites on the ZIF-8 surface (Figure 1).

The calculation performed for the neutral tetracoordinated optimized cluster [Zn^+2^(mIm^–^)_4_(H^+^)_2_] with QM options B3LYP/aug-cc-pVDZ/CHELPG/r(Zn)=0.95 Å yielded the best comparison with the bulk force field atomic charges^52^ prioritizing the Zn and N atoms: *q*(Zn) = 1.036*e* for our QM calculation and *q*(Zn) = 1.000*e* for the force field and *q*(N) = −0.472*e* for the average of two protonated and two deprotonated N atom not bonded to the Zn (N11) in our QM calculation and *q*(N) = −0.500*e* for the force field (see the atomic charges for all atoms in Table SI–S2). Therefore, we used the same QM options to analyze the atomic charges differences in the surface situation, i.e., optimized clusters with mono and dicoordinated Zn^+2^ atom with imidazole groups and with gas molecules to complete its tetracoordination: [Zn^+2^ mIm^–^]+(X_gas_)_3_ and [Zn^+2^(mIm^–^)_2_]+(X_gas_)_2_, with X_gas_ = CO_2_ or H_2_O. The optimized geometries and the atomic charges are shown in the SI (Figure S4 and Table S2). There is an increase of the atomic charge changing the coordination of Zn^+2^ atom (surface sites) compared with the tetracoodinated (bulk sites) from *q*(Zn) = +1.0e to +1.385e coordinated with one imidazole group [Zn^+2^ mIm^–^] and three CO_2_ molecules and +1.480*e* coordinated with two imidazole groups [Zn^+2^(mIm^–^)_2_] and two (CO_2_) molecules. For H_2_O molecules, those atomic charges increase to +1.614*e* and +1.691*e*, respectively. As expected, the polarization of the Zn atom was stronger for H_2_O (polar) molecules than for CO_2_ (nonpolar) molecules. However, considering the overall effect the atomic charge of the bulk Zn sites should increase from *q*(Zn) = +1.0*e* to +1.4–1.7*e* for surface Zn sites. These results show that the addition of excess charge to the unsaturated Zn atoms, i.e., assigning values of ca. +1.50∼+1.75*e* (see Table 2) is an adequate procedure. Moreover, the Zn atoms coordinated with one or two imidazole groups had similar charges for the same gas molecules. For this reason, the same atomic charges were assigned to unsaturated Zn atoms regardless of its coordination with one or two imidazole groups. On the account of these calculations, we have obtained the final set of atomic charges for the surface sites presented in Table 2.

### 3.2 Adequacy of MC Simulation Conditions to Represent Atmospheric Gases in Different Ensembles

**Figure 4:**
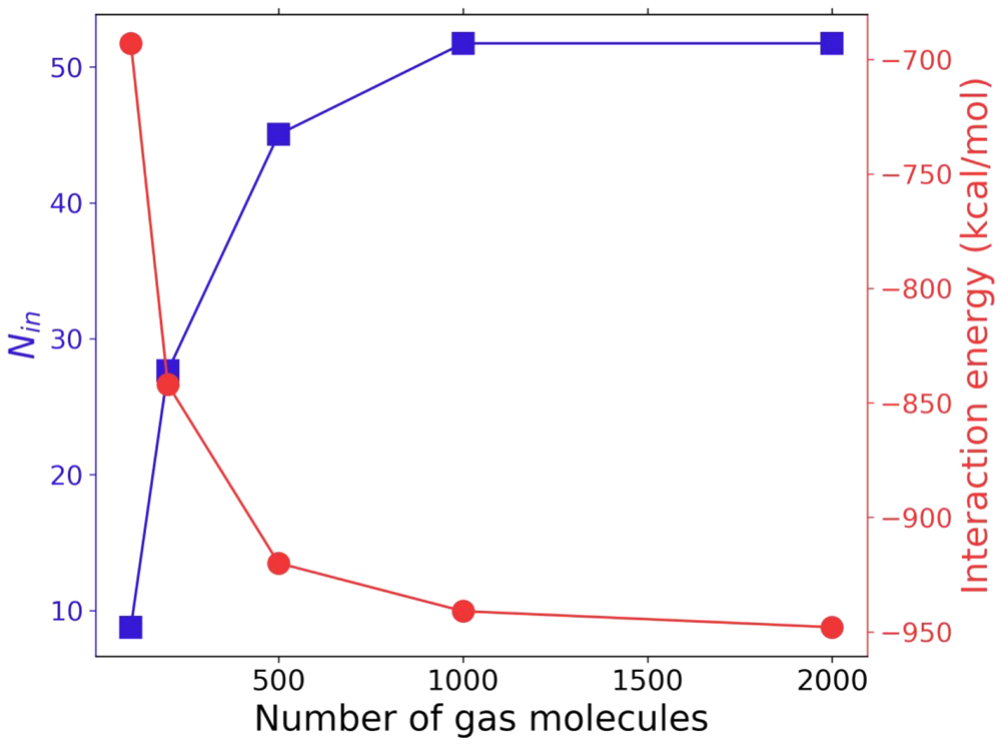
Convergence of the average interaction energy between the ZIF-8 and the gas molecules and the number of gas molecules within the nanoparticle pores, *N*_in_, as a function of the total number of gas molecules in the system, *N*_gas._ MC simulations were performed for system [(Zn^+2^)_24_(mIm^–^)_60_(Zn^+2^)_24_] and different amounts of CO_2_ molecules at constant ideal gas density and temperature of 273 K.

The MC simulations with a 1 × 1 × 1 ZIF-8 cubic nanoparticle required a large number of gas molecules to obtain the asymptotic behavior of atmospheric gas. However, to maintain atmospheric pressure, the box also had to be exceedingly large. We have dealt with this issue by varying the amounts of CO_2_ molecules and analyzing the convergence of the interaction energy between de ZIF-8 and the gas molecules and the number of gas molecules captured inside the nanoparticle pores, *N*_in_, as defined in Figure 3 from the center-of-mass up to 14.7 Å. Hence, the ZIF-8 nanoparticle was simulated in the NVT ensemble with N varying from 100 to 2000 of CO_2_ molecules at *T* = 273 K, *P* = 1 atm and different volumes *V* based on the density ρ of ideal gas (ρ = *N*/*V* = *P*/*kT*, where *k* is the Boltzmann constant). In these systems, the average interaction energy and the *N*_*in*_ barely changed in the interval between 1000 and 2000 gas molecules (Figure 4). For this reason, 1000 gas molecules were considered an adequate balance between the desired asymptotic behavior of the system and the computational cost for the simulations. Therefore, the Monte Carlo simulations of ZIF-8 nanoparticle described afterward were performed in the presence of 1000 gas molecules.

Furthermore, MC simulations of the ZIF-8 nanoparticle in the NPT ensemble were performed to examine whether the classical force field parameters can reproduce gas density without fixing the volume. The system was composed of one ZIF-8 1 × 1 × 1 nanoparticle with 1000 CO_2_ molecules under atmospheric pressure and at 273 K. The overall results, such as interaction energies and number of captured gas molecules, were similar to the NVT scenario. The main difference was the length of the cubic simulation box, which changed from 333.9 Å (NVT) to 307.0 Å (NPT). Because the NVT box size was chosen to reproduce the CO_2_ ideal gas density without the ZIF-8 nanoparticle, and the latter attracts CO_2_ molecules increasing the gas density compared to the ideal gas. Therefore, the volume reduction was expected.

### 3.3 Validation of Binding Energies and Geometries from MC Simulations against BOMD Simulations

**Figure 5:**
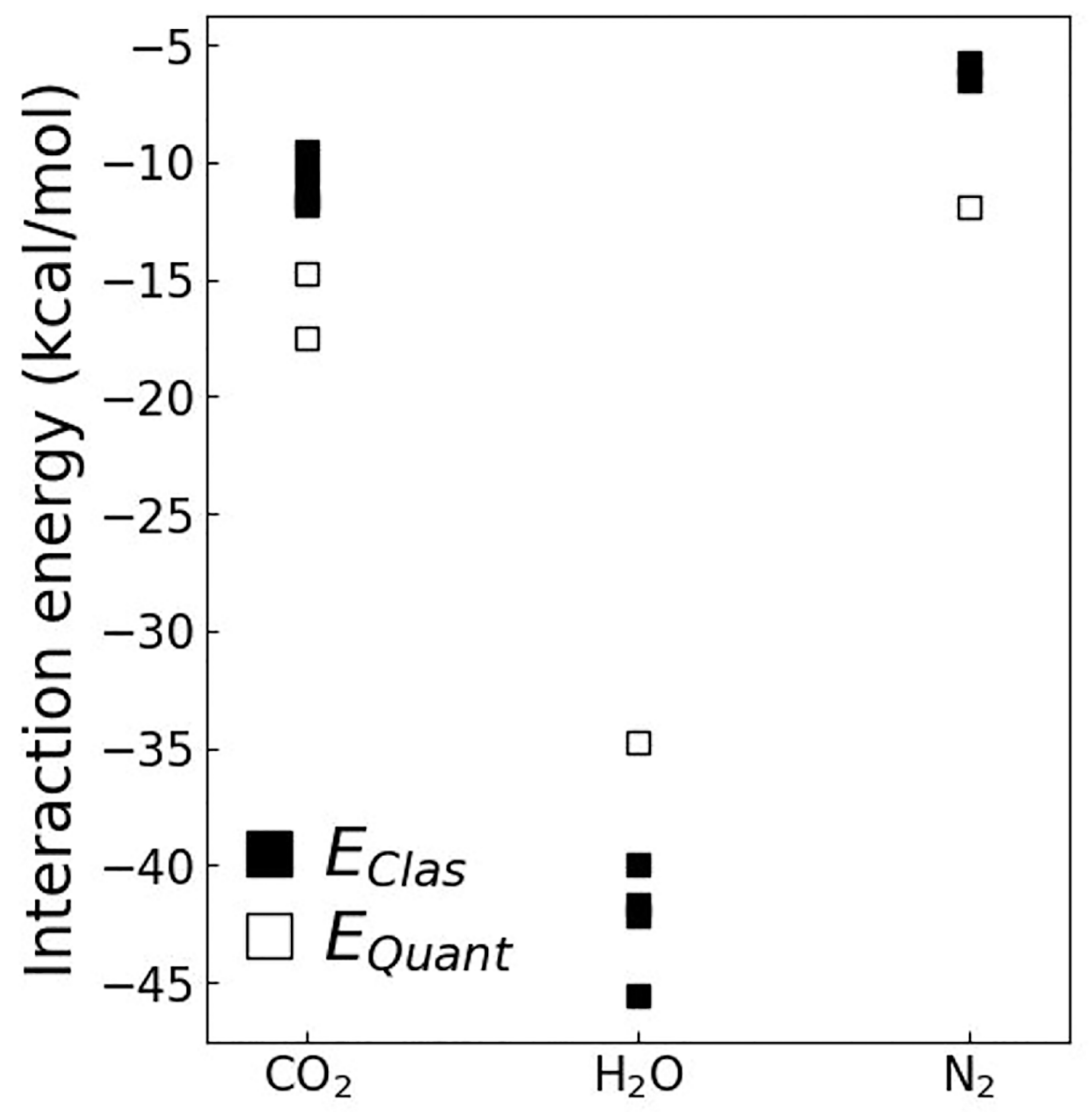
Comparison of average binding energy (⟨*E*_*ij*_⟩ in kcal/mol) between [Zn^+2^mIm^–^] and gas molecules (CO_2_, H_2_O, and N_2_) calculated using the configurations generated with MC and BOMD simulations and with optimized clusters (energies presented in Table 3 and geometries in Figure 6). The closed symbols are assigned to classical force field energies, and open symbols represent B3LYP/cc-pVDZ ones.

The average binding energy, ⟨*E*_*ij*_⟩, calculated with the classical force field (FF) and the distance between an unsaturated Zn atom from ZIF-8 and CO_2_, H_2_O, or N_2_ molecules, ⟨*R*_Zn-X_⟩, were compared for configurations generated with (i) classical Monte Carlo simulations at atmospheric pressure and 273 K (MC), (ii) geometry optimized clusters (OPT), and (iii) Born–Oppenheimer molecular dynamics simulations at 298 K (BOMD) (Table 3). In the MC, deep local minima were present in the RDF between Zn and O at 3.25 Å for CO_2_, at 2.75 Å for H_2_O, and between Zn and N at 2.95 Å for N_2_ (Figure SI–S7). Thus, these values were used as the cut distances of interacting gas molecules with the surface sites. For the optimized clusters, the binding energies ⟨*E*_*ij*_⟩ were also calculated with QM method and compared with the values obtained with the classical FF (Table 3 and Figure 5). The geometries for the [Zn^+2^mIm^–^] systems interacting with N_2_, CO_2_, and H_2_O molecules are presented in Figure S5.

**Table 3:** Average Binding Energies (⟨*E*_*ij*_⟩ in kcal/mol) Calculated with the Classical Force Field for Configurations Generated with MC and BOMD Simulations and for the Geometry Optimized Cluster and Average Distances (⟨*R*_Zn-X_⟩ in Å) between the Surface Zn Atom and the Nearest Atom of the Gas Molecule^a^

			CO_2_		H_2_O		N_2_	
method	solute	*N*_gas_	⟨*E*_*ij*_⟩	⟨*R*_Zn–O_⟩	⟨*E*_*ij*_⟩	⟨*R*_Zn–O_⟩	⟨*E*_*ij*_⟩	⟨*R*_Zn–N_⟩
MC^b^	[(Zn^+2^)_24_(mIm^–^)_60_(Zn^+2^)_24_]	1000	–15.1	2.21	–62.2	1.93	–8.4	2.26
MC^b^	[Zn^+2^mIm^–^]	100	–11.5	2.18	–45.5	1.98	–5.7	2.22
OPT	[Zn^+2^mIm^–^]	3	–11.8 (−17.5)	2.12	–41.7 (−34.7)	2.08	–6.5 (−11.9)	2.21
OPT	[Zn^+2^mIm^–^]	4	–10.5 (−14.7)	2.26	–42.1	2.05	−	−
BOMD^b^	[Zn^+2^mIm^–^]	4	–9.5	2.21	–40.0	2.09	−	−

			⟨*E*_*ij*_⟩	⟨*R*_H–O_⟩	⟨*E*_*ij*_⟩	⟨*R*_H–O_⟩	⟨*E*_*ij*_⟩	⟨*R*_H–N_⟩
OPT	[mImH]	1	–2.3 (−1.5)	2.28	–7.5 (−5.8)	1.99	–1.3 (−1.0)	2.46

a For comparison, it is also shown in parentheses the binding energies calculated with QM calculations with B3LYP/cc-pVDZ.

b Average over 5000 configurations generated in the simulations.

The distances between the Zn atoms of the [(Zn^+2^)_24_(mIm^–^)_60_(Zn^+2^)_24_] nanoparticle surface, or of the [Zn^+2^mIm^–^] cluster, and the oxygen atoms of the CO_2_ and H_2_O molecules, ⟨*R*_Zn–O_⟩, and nitrogen atoms N_2_, ⟨*R*_Zn–N_⟩, present similar values independently of the method (classical simulation, *ab initio* simulation and optimized clusters) used to generate the conformations/geometries (Table 3). The average values of ⟨*R*_Zn–O_⟩ varies from 2.12 to 2.26 Å for CO_2_, and from 1.93 to 2.09 Å for H_2_O molecules, and ⟨*R*_Zn–N_⟩ varies from 2.21 to 2.26 Å for N_2_ molecules. This ensures that the force field parameters describe adequately the interactions of the nanoparticle surface sites with the gas molecules. The distances between the H atom of the mImH group and the oxygen atoms of the CO_2_ and H_2_O molecules, ⟨*R*_H–O_⟩, and the nitrogen atoms of the N_2_, ⟨*R*_H–N_⟩, obtained for optimized clusters show the occurrence of hydrogen bonds (H-bond) with distances of 2.28 Å for CO_2_, 1.99 Å for H_2_O and 2.46 Å for N_2_. As expected, the H_2_O presents a shorter H-bond with a binding energy of −7.5 kcal/mol calculated with the classical FF and −5.8 kcal/mol with QM calculation. The N_2_ forms a longer H-bonds with a weaker binding energy of −1.3 kcal/mol calculated with the classical FF, and −1.0 kcal/mol calculated with the QM method. The QM and classical FF binding energies are within the same order of magnitude (Table 3 and Figure 5), indicative of the consistence of the classical FF parameters with the QM interactions. It is noteworthy that the binding energies between [mImH] and CO_2_ or N_2_ are comparable to *kT*, which means that thermal effects may disrupt the capture of these gas molecules in the surface sites containing protonated imidazolate groups (Figure 1).

The analysis of ⟨*E*_*ij*_⟩ between the 1 × 1 × 1 ZIF-8 nanoparticle and the gas molecules from configurations generated through MC simulations shows a stronger interaction of −62.2 kcal/mol with H_2_O, then −15.1 kcal/mol with CO_2_ and −8.4 kcal/mol with N_2_. Clearly, the H_2_O molecules are much more attracted to the ZIF-8 surface than the CO_2_ and N_2_ gases. However, performing the same analysis for a smaller system, [Zn^+2^mIm^–^], the values of ⟨*E*_*ij*_⟩ become −45.5 kcal/mol for H_2_O, −11.5 kcal/mol for CO_2_ and −5.7 kcal/mol for N_2_, a significant decrease of the interaction energy for the H_2_O molecules but less so for the CO_2_ and N_2_ gases. This pattern indicates that the attractive interactions of CO_2_ and N_2_ molecules with the ZIF-8 are more local and short-range than the interaction with H_2_O molecules, possibly for the stronger Coulombic interaction with the water molecules and the entire nanoparticle structure that causes an attractive long-range interaction. It also indicates that a potential cooperative effect among gas molecules is not important as the ⟨*E*_*ij*_⟩ values present small variations with respect to *N*_gas_. For example, ⟨*E*_*ij*_⟩ varies from −11.5 kcal/mol (or −45.5 kcal/mol) for 100 CO_2_ (or H_2_O) to −11.8 kcal/mol (or −41.7 kcal/mol) for 3 CO_2_ (or H_2_O) molecules.

### 3.4 The Effect of Surface Chemical Composition, Temperature, and Pressure on the Competitive Adsorption of Atmospheric Gases

A set of MC simulations was performed for the 1 × 1 × 1 ZIF-8 in binary mixtures of the most abundant atmospheric gases (Table 4). The affinity for CO_2_ molecules inside of ZIF-8 greatly surpasses the affinities of any of the considered gases. Remarkably, H_2_O molecules were captured exclusively on the nanoparticle surface, being fully absent inside of ZIF-8 pores. This is in agreement to what was expected from the assumed hydrophobicity of the framework pores.^35,37^ Therefore, there is a clear pattern of gas capture with all CO_2_ molecules bound inside the framework pores and all the H_2_O molecules bound at the material surface (Table 4). This finding is consistent with previous experimental measurements where a combination of XPS and TPD was employed to differentiate the absorption of CO_2_ from the adsorption of H_2_O.^50^ The present simulations also show a much greater uptake of CO_2_ compared to N_2_ at atmospheric pressure than previously observed via absorption measurements of CO_2_:N_2_ mixtures by ZIF-8.^9^ The least interacting gases, O_2_ and Ar, can only be captured by ZIF-8 at temperatures below 77 K.^15,71^ Hence, the CO_2_ capture is unparalleled inside of ZIF-8 though H_2_O molecules have a more favorable interaction energy with the nanoparticle surface when compared to CO_2_ and N_2_ molecules (Table 3).

**Table 4:** Adsorption of Pristine Gases to ZIF-8 Nanoparticles under Atmospheric Pressure and Temperature of 273 K^a^

gas	*N*_in_	*N*_surf_	*N*_int_	⟨*E*_surf_/*N*_surf_⟩	⟨*E*/*N*_int_⟩
H_2_O	0	85	577	–62.2	–6.7
CO_2_	52	103	196	–15.1	–4.8
N_2_	2	49	57	–8.4	–3.6
O_2_	2	0	8	–1.9	–0.2
Ar	1	1	2	–0.8	–0.7

a *N*_in_ and *N*_surf_ are the number of gas molecules inside and at the surface of ZIF-8, *N*_int_ is the number of gas molecules within an interaction range, i.e., with the binding energy stronger than −0.05 kcal/mol, ⟨*E*_surf_/*N*_surf_⟩ is the average binding energy per gas molecule at the surface of the ZIF-8 and ⟨*E*/*N*_int_⟩ is average interaction energy per gas molecule. Energies are in kcal/mol.

To account for surface composition of the ZIF-8 nanoparticle due to variable synthesis conditions, different Zn coordination states have been considered in our simulations. These states are associated with distinct nanoparticle charges, which may enhance or decrease CO_2_ capture. Three compositions of the surface sites, Zn^+2^ sites, protonated imidazolates H^+^, and bare nitrogen deprotonated imidazolates mIm^–^, were represented in the MC simulations (Figure 1). The assigned atomic charges are presented in Table 2.

**Figure 6:**
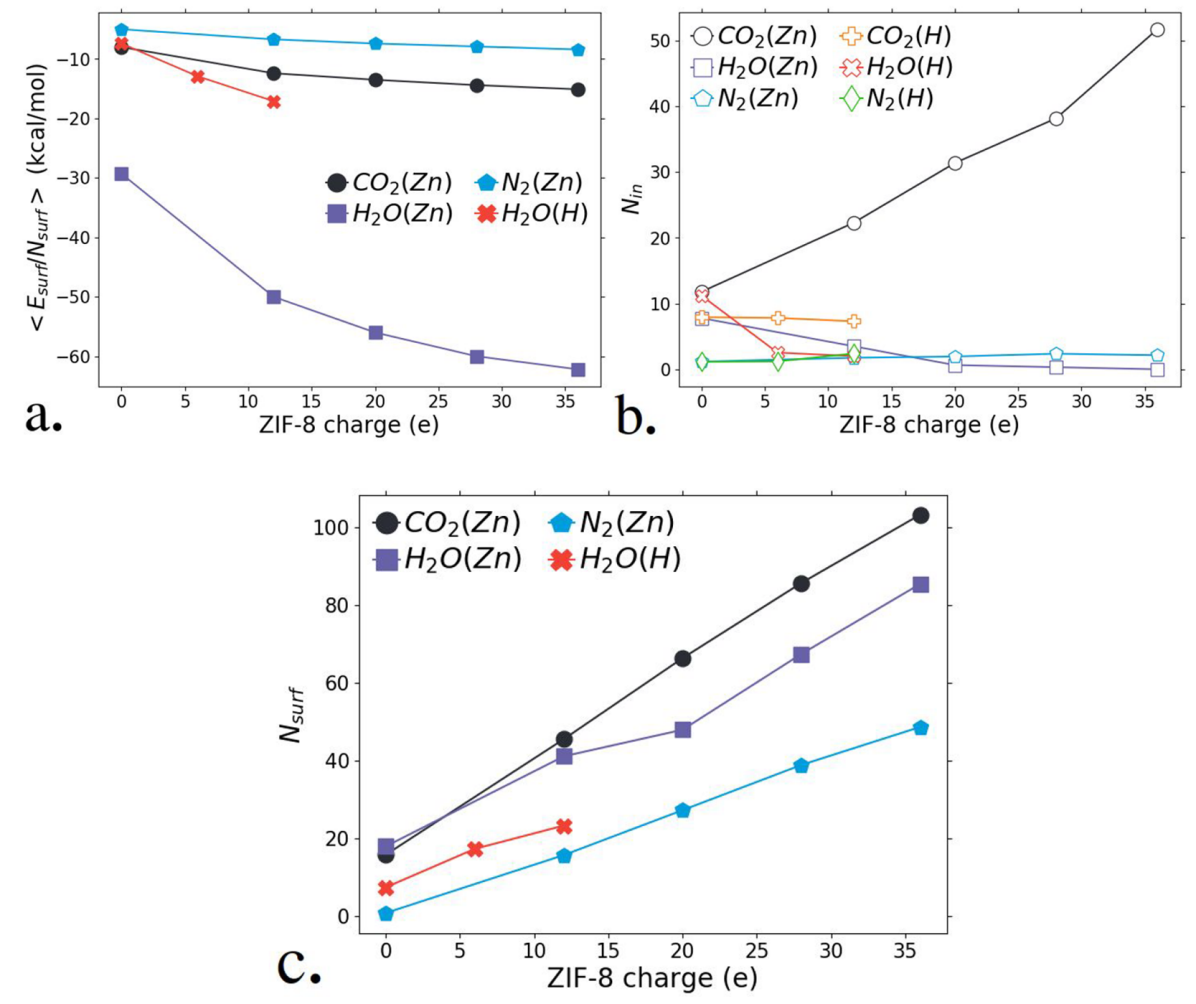
(a) Average surface interaction energy per gas molecule, ⟨*E*_surf_*N*_surf_⟩ and (b) number of CO_2_, N_2_ and H_2_O molecules captured in the nanoparticle pores (*N*_in_) or (c) at the surface (*N*_surf_) as a function of the total surface charge. The representations (Zn) and (H) refer to the composition of the surface sites, X_surf_ = Zn^+2^ or H^+^, respectively.

The MC simulations show that CO_2_ or N_2_ gases are not captured on the ZIF-8 nanoparticle surface when it is represented by protonated imidazolates binding sites. The MC-derived average complexation energies are −2.3 (CO_2_), −1.3 (N_2_), and −7.5 (H_2_O) kcal/mol (Table 3) which are consistent with the QM-single point energy calculations of [mImH] complexed with CO_2_, N_2_, and H_2_O molecules, −1.5, −1.0, and −5.8 kcal/mol, respectively. These findings imply that the intermolecular interactions between the protonated imidazolate group NH and the two gases are easily disrupted by thermal effects whereas interactions with H_2_O are significantly more favorable. Although the capture of CO_2_ is favored over N_2_, H_2_O poses a strong competition to the capture of atmospheric CO_2_ in all investigated surface configurations (Figure 6a). Yet, the capture of CO_2_ inside the ZIF-8 is unmatched by H_2_O (Figure 6b). The presence of Zn^+2^ atoms on the surface of the nanoparticle greatly improves CO_2_ capture inside of ZIF-8. On the other hand, the increase of surface charges, via protonation of the imidazolate linker, either decreases or does not impact CO_2_ capture inside of the framework. Therefore, CO_2_ capture is greatly improved if the ZIF-8 nanoparticle has a higher Zn weight ratio, though not necessarily a higher charge. Our findings indicate that larger ZIF-8 nanoparticles (i.e., smaller surface to volume ratios) will allow the capture of larger amounts of atmospheric CO_2_. However, the same findings show that smaller ZIF-8 nanoparticles provide a larger number of bare Zn^+2^ surface sites for which H_2_O exhibits decreased binding affinity. Consequently, the optimal size of a ZIF-8 nanoparticle for efficient CO_2_ capture depends on the balance between low surface to volume ratio and a surface richer in protonated imidazolate binding sites.

The competitive adsorption of the most abundant atmospheric gases has also been investigated via MC simulations of the 1 × 1 × 1 ZIF-8 nanoparticle in the presence of CO_2_:H_2_O and CO_2_:N_2_. In these systems, the surface binding sites were represented by bare Zn^+2^ atoms (Figure 1). For CO_2_:H_2_O mixtures, the surface capture was dominated by H_2_O molecules due to the more favorable interaction energy compared with CO_2_. In these atmospheric-like conditions, 9 CO_2_ molecules on average were captured inside of the nanoparticle compared to 52 CO_2_ molecules in the pristine gas simulations. According to Liu et al.,^48^ the CO_2_ capture by ZIF-8 nanoparticles can be enhanced in a humid media. On the other hand, exposure of ZIF-8 to acid atmospheric conditions leads to irreversible structural degradation, the more so as the relative humidity of the environment increases.^47,72^ Our findings indicate that a smaller surface-to-volume ratio of ZIF-8 nanoparticles can increase the capture of CO_2_ relative to H_2_O while decreasing the substantial losses in crystallinity and textural properties of ZIF-8. On the other hand, there is a clear preference for the capture of CO_2_ over N_2_ by the ZIF-8 nanoparticle in gaseous mixtures of equal amounts of CO_2_:N_2_ molecules. On average, 34 CO_2_ versus 1 N_2_ molecules are captured inside of the ZIF-8 pores. Further, N_2_ molecules were not observed to bind to the bare Zn^+2^ surface sites. Hence, the capture of CO_2_ in binary 1:1 mixture with N_2_ is lower than in pristine CO_2_ gas, with a decrease from 52 to 34 molecules, but it remains highly selective toward CO_2_.

A set of MD simulations with ZIF-8 nanoparticles of different sizes was performed to evaluate the surface effect, since bigger nanoparticles have smaller surface-to-volume ratios. The results are in Table 5. A bigger ZIF-8 nanoparticle exhibits higher CO_2_ uptake, see *N*_in_ and *N*_surf_, since more capture sites are available. However, this increase does not abide by the rhythm at which the ZIF-8 nanoparticle structure grows. It can be seen by the steep decaying tendency of both internal CO_2_ density and total loading as the nanoparticle size increases (i.e., as the surface-to-volume ratio diminishes). The values found for CO_2_ loading by ZIF-8 for atmospheric environment are within 22–60 and 8–82 mg/g, for experimental^9,10,73^ and theoretical studies,^11,24,39,44,74−78^ respectively. The experimental procedures consider the ZIF-8 in dimensions orders of magnitude larger than the ones in Table 5, and the theoretical works deal with a bulk replicated by periodic boundary conditions (i.e., the limit of zero surface-to-volume ratio). This means that the loadings in Table 5 were expected to be larger than the aforementioned values in view of the sharp loading decrease as the nanoparticle grows. Local surface capture factors such as CO_2_ coordination, binding energy and geometry with Zn were unchanged between different nanoparticles.

**Table 5:** Adsorption of CO_2_ to ZIF-8 Nanoparticles under Atmospheric Pressure and Temperature of 273 K^a^

ZIF-8 nanoparticle size	*N*_in_	*N*_surf_	internal CO_2_ density (g/cm^3^)	total loading (mg/g)
1 × 1 × 1	117	120	0.657	1304
2 × 2 × 2	195	322	0.210	550
3 × 3 × 3	434	605	0.161	393

a *N*_in_ and *N*_surf_ are the number of gas molecules inside and at the surface of ZIF-8. Internal CO_2_ density is regarding *N*_in_, while total loading considers both *N*_in_ and *N*_surf_.

For the most part, we have chosen to simulate a unit cell representation of the ZIF-8 nanoparticle for which surface defects can be fully neglected. Our approach provides a simpler, but highly controllable, structural representation of ZIF-8 that can be contrasted to experimental adsorption measurements where such structural homogeneity is not easily attained. It has been previously shown that the effect of outer surface area of ZIF-8 crystals on water sorption is non-negligible as crystals become smaller.^37^ These measurements of H_2_O uptake by ZIF-8 were performed at relative pressures of 0.95 and 0.1. It was observed an increase in H_2_O uptake with the increase in the ZIF-8 crystal dimensions (i.e., 324, 15.8, and 0.4 μm) whereas the heat of adsorption decreased with increasing sorption amounts before becoming constant.^37^ These observations have been interpreted as due to the sorption inside ZIF-8 pores combined with the existence of structural defects where higher interaction energy with H_2_O molecules occurs. However, H_2_O sorption inside of ZIF-8 pores cannot be easily conciliated with our calculations where H_2_O strongly favors the binding to surface sites as opposed to the inner pores of ZIF-8 (Figure 6). The computational simulations and experimental measurements can, however, be conciliated if the ZIF-8 surface defects in the nanoparticles used in the experimental is more extensive than suspected. This is clearly a point that will need the development and implementation of capabilities for the atomic-scale detection of defects in ZIFs.

We have also investigated the role of temperature and pressure for the selective capture of atmospheric CO_2_. MC simulations of the ZIF-8 nanoparticle were performed at 298 and 273 K under 1 atm of pressure, and at 273 K and under 20 atm (Table 6). The different temperatures had no noticeable effect on the intermolecular interaction energies from the simulated systems. However, the number of molecules captured decreased systematically with the increase of the temperature. This is consistent with measurements of pure CO_2_ sorption at 273, 298, 323, and 348 K which described a severe decrease of the amount of captured CO_2_ by ZIF-8 upon temperature increase.^9^ Remarkably, lower temperatures amplified the CO_2_ capture in both extremes of charge configurations, that is, fully positive (+36*e*) and neutral (0*e*) (Table 6). In contrast, H_2_O capture increases at higher temperatures (Table 6). This effect can be explained because of the increase in the kinetic energy of the water molecules that enlarge their movement in the space and eventually find the long-range attractive interaction of the ZIF-8. Therefore, enhanced selective capture of atmospheric CO_2_ against H_2_O by ZIF-8 nanoparticles can be attained through the modulation of temperature and the Zn weight ratio in the particles.

**Table 6:** Effects of Temperature and Pressure on Atmospheric CO_2_ Capture^a^

		*N*_in_		*N*_surf_		*N*_int_		*E*_surf_/*N*_surf_		*E*/*N*_int_	
gas	*Q*(*e*)	298 K	273 K	298 K	273 K	298 K	273 K	298 K	273 K	298 K	273 K
CO_2_	36	30	52 (108)	95	103 (107)	160	196 (719)	–15.4	–15.1 (−15.2)	–5.1	–4.8 (−1.8)
CO_2_	0	7	12	14	16	25	32	–7.9	–8.0	–2.8	–2.8
H_2_O	36	0	0	94	85	588	577	–61.6	–62.2	–7.1	–6.7

a MC simulations were performed for the ZIF-8 nanoparticle containing bare Zn atoms as surface binding site under different conditions of temperature and pressure of 1 atm. CO_2_ capture is presented in terms of the number of gas molecules bound to the ZIF-8 surface (*N*_surf_), inside its pores (*N*_in_) and with binding energies stronger than −0.05 kcal/mol (*N*_int_) of the ZIF-8 nanoparticle. The values in parentheses are from MC simulations with 20 atm.

The pressure affects severely the amount of CO_2_ adsorbed to the ZIF-8 nanoparticle (Table 6). The MC simulation with all 24 bare Zn surface sites at 273 K and 20 atm showed an increase of CO_2_ capture as measured by the increase of *N*_in_, *N*_surf_ and ⟨*E*_surf_/*N*_surf_⟩ for this gas (Table 6). It should be noticed that CO_2_ remained in the gas state without the use of any type of constraints over the simulation box volume, attesting to the quality of the atomic parameters used in our classical simulations. Remarkably, the change of the pressure from 1 to 20 atm more than doubled *N*_in_, while it barely affected *N*_surf_ for CO_2_ molecules (Table 6). This is qualitatively consistent with experimental measurements, which have shown an 8-fold increase of the amount of adsorbed CO_2_ by ZIF-8 nanoparticles at 20 atm.^10^ The discrepancy between the calculated and experimental estimates can be explained by the small size of the simulated nanoparticle. Since the pressure effect is mostly expressed as the capture of CO_2_ inside of the ZIF-8 nanoparticle, the single cell unit of only 1 × 1 × 1 used in the MC simulations has a high surface-to-volume ratio, leading to a less pronounced increase of CO_2_ capture at 20 atm. However, there is an unmistakable trend toward enhanced CO_2_ capture at higher pressures, especially if combined with smaller surface-to-volume ratios.

## 4 Conclusion

The framework surface is the first point of interaction between the porous adsorbent and the adsorbate. This interaction becomes more critical for particles at the nanoscale level so that an adequate description of such processes requires models and methods that account for the chemical structure and composition of the framework surface. This can be attained via atomistic simulations of nanoparticles combining different boundary structures and accurate interaction potentials. We have performed atomistic simulations across the quantum and mechanical chemistry scales to ascertain the role of surface binding site composition on the selective capture of atmospheric CO_2_ by a ZIF-8 nanoparticle in pristine (CO_2_, N_2_, O_2_, Ar, H_2_O) and binary (CO_2_:N_2_, CO_2_:H_2_O) mixtures. Classical force-field parameters were successfully refined against QM calculations, BOMD and experimental data to reproduce the geometry and binding interactions of different binding site compositions in the ZIF-8 nanoparticle with atmospheric gases.

We have found that for all surface binding site representations, except the neutral one with H atoms, the selectivity toward CO_2_ is the highest among the most abundant atmospheric gases at the inner cavities of the ZIF-8 nanoparticle. The boundary interface can capture CO_2_, H_2_O and N_2_, though the interaction energy is strongly more favorable for H_2_O. Although the presence of H_2_O molecules hinders the interaction between ZIF-8 nanoparticles and CO_2_, the presence of N_2_ molecules barely affects the CO_2_ adsorption. Remarkably, CO_2_ capture increases for nanoparticles richer in surface unsaturated Zn atoms and in smaller nanoparticles. Furthermore, the thermodynamics conditions evaluated, temperature and pressure, have negligible effect on the interaction energies, though the number of gas molecules captured are strongly dependent on both thermodynamical properties. Hence, lower temperatures and higher pressures aid positively the CO_2_ adsorption.

The present findings underscore the importance of accounting for a boundary interface and surface chemical composition in atomistic simulations of selective gas adsorption by metal–organic-framework nanoparticles. This approach indicates that ZIF-8 nanoparticles with a higher surface-to-volume ratio and high Zn weight ratio will exhibit enhanced selectivity for atmospheric CO_2_ capture. Hence, the optimal size of a ZIF-8 nanoparticle for efficient CO_2_ capture will depend on high surface-to-volume ratio and a surface richer in protonated imidazolate binding sites. Since ZIF-8 is susceptible to irreversible surface structural degradation with the increase of the relative humidity of acid atmospheric environments, a straightforward drying/dehydration pretreatment of the stream to remove water vapor offers an economically viable option. Furthermore, the demonstration that H_2_O molecules bind significantly more to the ZIF-8 surface than to the inner pore invites to a new interpretation of previous measurements of H_2_O sorption by ZIF-8 nanocrystals of different dimensions.

## Data Availability

Input files, topologies, atomic parameters, and initial coordinates for Monte Carlo (MC), molecular dynamics (MD), Born–Oppenheimer molecular dynamics (BOMD) simulations of ZIF-8 accounting for different chemical composition of the nanoparticle surface and unit cells are freely available for download (10.5281/zenodo.6527693)

## Supporting Information Available

Structural analysis plus cutoff distances used for that end, force field parameters, list of simulated systems via Monte Carlo, molecular dynamics, and Born–Oppenheimer molecular dynamics (PDF)

## Notes

As female scientists in the so-called hard sciences, the authors have experienced, and witnessed, the difficulties faced by minorities in academia. The authors are fully committed to supporting and promoting the advancement of women, LGBTQ+, and geographical minorities in chemistry.
