# Supplementary material for: The Effect of Surface Composition on the Selective Capture of Atmospheric CO2 by ZIF Nanoparticles: The Case of ZIF-8
Source: J Chem Inf Model. 2022 Sep 23;62(24):6530–43. doi: 10.1021/acs.jcim.2c00579 (PMC9795550; doi:10.1021/acs.jcim.2c00579)
Supplement: Supplementary file 1 — ci2c00579_si_001.pdf [file ci2c00579_si_001.pdf]

SUPPLEMENTARY INFORMATION

The Effect of Surface Composition on the Selective Capture of Atmospheric CO_2 by ZIF Nanoparticles: The case of ZIF-8

Alexsander C. Vendite†, Thereza A. Soares†§ and Kaline Coutinho†**

†Instituto de Física, Universidade de São Paulo, Cidade Universitária,

São Paulo 05508-090, Brazil

§Hylleraas Centre for Quantum Molecular Sciences, University of Oslo,
0315 Oslo, Norway

S1. ZIF-8 Force Field Parameters (Figure S1 and S2, and Table S1)
S2. Atomic Charges (Figure S3 and S4, and Table S2)
S3. Optimized [$Zn^{+2}mIm^{-}$] Clusters with CO_2, H_2O and N_2 (Figure S5)
S4. Simulated Systems (Table S3, S4 and S5)
S5. ZIF-8 Flexibility (Figure S6)
S6. Solvation of the ZIF-8 Nanoparticle (Figure S7)

S1. ZIF-8 Force Field Parameters

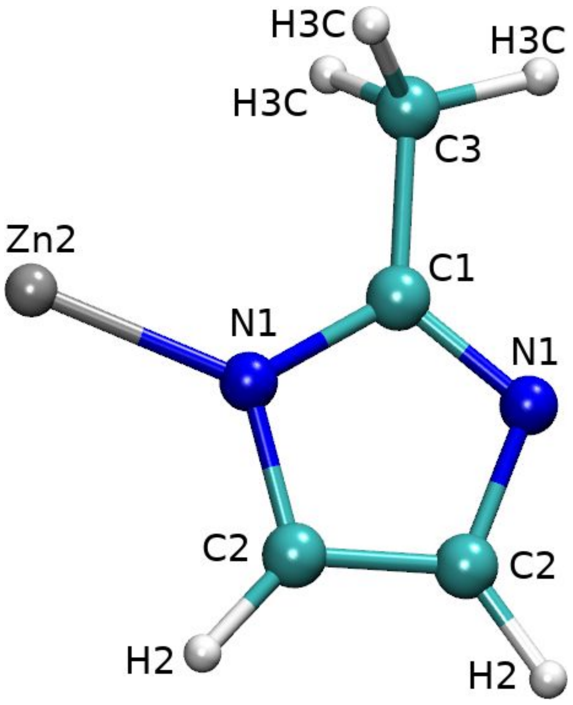

Figure S1: Molecular representation and labels of the [Zn^{+2} mIm^{-}] group used in the force field parameters list.

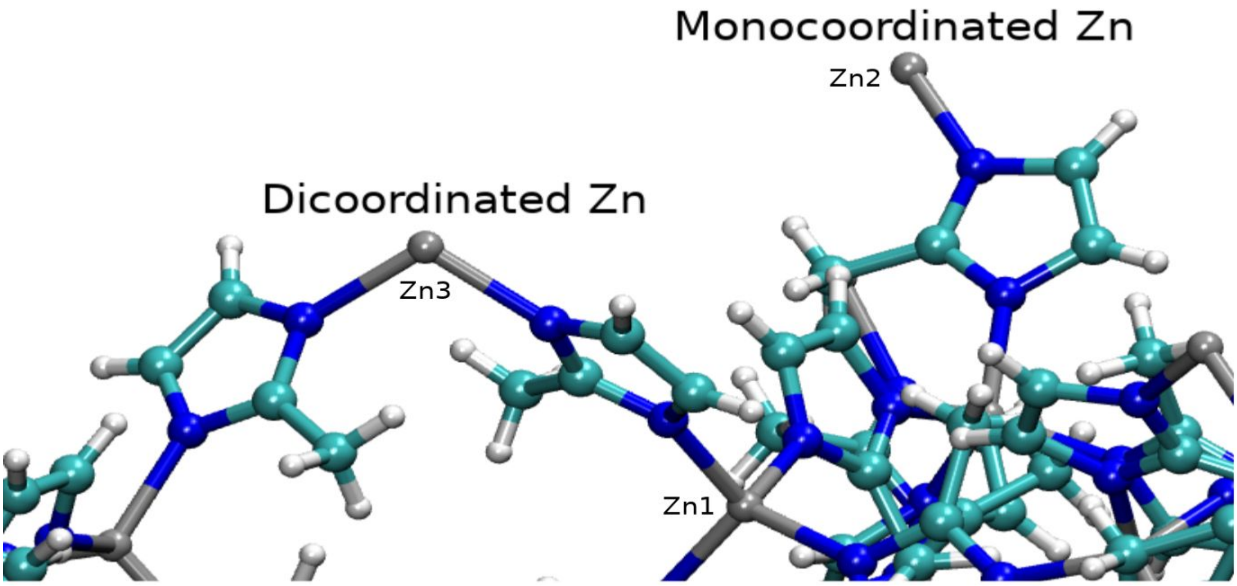

Figure S2: Molecular representation of the ZIF-8 nanoparticle surface region, for 2x2x2 and 3x3x3 nanoparticles, that present saturated bulk Zn atoms coordinated with four imidazole groups (Zn1, tetracoordinated), unsaturated surface Zn atoms coordinated with one imidazole group (Zn2, monocoordinated) and coordinated with two imidazole groups (Zn3, dicoordinated). The 1x1x1 ZIF-8 nanoparticle has only monocoordinated *Zn* atoms in the surface.

Table S1: List of the non-bonded and bonded force field parameters taken from AMBER force field previously adapted for ZIF-8 by Hu et al.[1] and refined by us, presented in unit of the DICE program.[2] The atomic charges of the unsaturated surface Zn atoms (showed in Figure S2) were described in the main text (see Table 2) because it depends on the three different chemical moieties $(X_{surf})_n$ used in the termination of the surface.

#atom	q(*e*)	ε(kcal/mol)	σ(Å)	label for the bond, angle, dihedral and improper
Zn1	1.00	0.0125	1.960	Zn1
Zn2	see_text	0.0125	1.960	Zn2
Zn3	see_text	0.0125	1.960	Zn1
N1	-0.50	0.1700	3.250	N
C1	0.50	0.0860	3.400	C1
C2	-0.10	0.0860	3.400	C2
C3	-0.30	0.1094	3.400	C3
H2	0.10	0.0150	2.421	H2
H3C	0.10	0.0157	2.650	H3C

$bond

#bond	K_r(kcal/mol Å^2)	r_{eq}(Å)
Zn1-N	83.6520	1.987
N-C1	488.002	1.340
N-C2	410.002	1.371
C1-C3	317.006	1.493
C2-C2	517.997	1.346
C2-H2	366.993	0.929
C3-H3C	339.998	0.960
Zn2-N	83.6520	1.987

$end bond

$angle

#angle	type	K_θ(kcal/mol Å^2)	θ_{eq}(degree)
N-Zn1-N	harmonic	11.950	109.47
Zn1-N-C1	harmonic	20.316	128.35
Zn1-N-C2	harmonic	21.510	126.40
C1-N-C2	harmonic	70.000	105.24
N-C1-N	harmonic	70.000	112.17
N-C1-C3	harmonic	70.000	123.89
N-C2-C2	harmonic	70.000	108.67
N-C2-H2	harmonic	50.000	125.66
C2-C2-H2	harmonic	50.000	125.67
C1-C3-H3C	harmonic	50.000	109.44
H3C-C3-H3C	harmonic	35.000	109.50
C1-N-Zn2	harmonic	20.316	128.35
C2-N-Zn2	harmonic	21.510	126.40

$end angle

$dihedral

#dihedral	format	F1	F2	F3(kcal/mol)	phase1	phase2	phase3(degree)
Zn1-N-C1-N	AMBER	0.000	15.000	0.000	0.000	180.000	0.000
Zn1-N-C1-C3	AMBER	0.000	15.000	0.000	0.000	180.000	0.000
C2-N-C1-N	AMBER	0.000	15.000	0.000	0.000	180.000	0.000
C2-N-C1-C3	AMBER	0.000	15.000	0.000	0.000	180.000	0.000
Zn1-N-C2-C2	AMBER	0.000	15.000	0.000	0.000	180.000	0.000

Zn1-N-C2-H2	AMBER	0.000	15.000	0.000	0.000	180.000	0.000
C1-N-C2-C2	AMBER	0.000	15.000	0.000	0.000	180.000	0.000
C1-N-C2-H2	AMBER	0.000	4.800	0.000	0.000	180.000	0.000
N-C2-C2-N	AMBER	0.000	15.000	0.000	0.000	180.000	0.000
N-C2-C2-H2	AMBER	0.000	4.000	0.000	0.000	180.000	0.000
H2-C2-C2-H2	AMBER	0.000	4.000	0.000	0.000	180.000	0.000
N-C1-C3-H3C	AMBER	0.000	0.000	0.100	0.000	0.000	0.000
C1-N-Zn1-N	AMBER	0.000	0.000	15.000	0.000	0.000	8.800
C2-N-Zn1-N	AMBER	0.000	0.000	15.000	0.000	0.000	-171.200
C2-C2-N-Zn2	AMBER	0.000	15.000	0.000	0.000	180.000	0.000
H2-C2-N-Zn2	AMBER	0.000	15.000	0.000	0.000	180.000	0.000
N-C1-N-Zn2	AMBER	0.000	15.000	0.000	0.000	180.000	0.000
C3-C1-N-Zn2	AMBER	0.000	15.000	0.000	0.000	180.000	0.000
$end dihedral							

$improper dihedral		
#dihedral	F1(kcal/mol)	phase1(degree)
N-Zn1-C1-C2	15.000	180.00
C1-N-N-C3	15.000	180.00
C2-N-C2-H2	8.500	180.00
N-C2-C1-Zn2	15.000	180.00
$end improper dihedral		

S2. Atomic charges

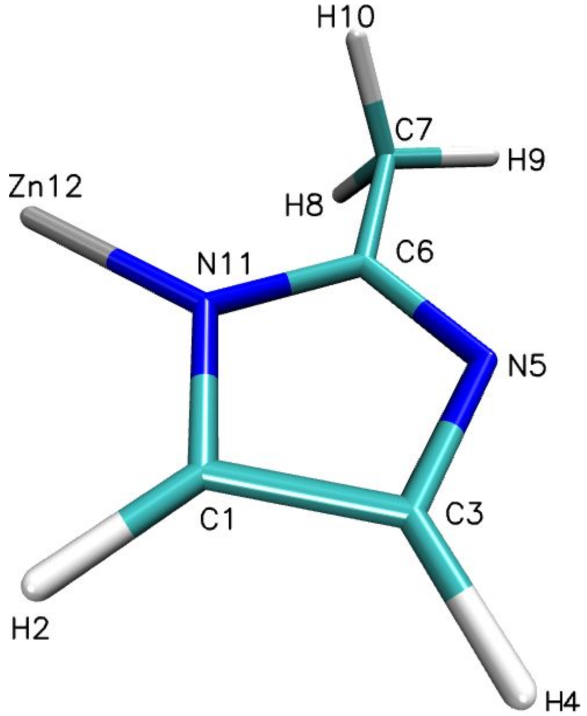

Figure S3: Molecular representation and labels of the [$Zn^{+2}(Im^{-})$]$^{+}$ group used in the quantum mechanics calculations showed in Table S1.

Table S2: Comparison of the atomic charges (averaged over equivalent atoms) calculated with quantum mechanics calculations using B3LYP/aug-cc-pVDZ and CHELPG for the fitting of the electrostatic potential using the van der Waals radius of Zn as 0.95 Å and the ZIF-8 bulk force field[1], for the following systems: (a) $[Zn^{+2}(mIm^{-})_4\,(H^{+})_2]$; (b) $[Zn^{+2}(Im^{-})_2]$+$(H_2O)_2$; (c) $[Zn^{+2}(Im^{-})_2]$+$(CO_2)_2$; (d) $[Zn^{+2}(mIm^{-})]^{+}$+$(H_2O)_3$; and (e) $[Zn^{+2}(mIm^{-})]^{+}$+$(CO_2)_3$, see optimized geometries in Figure S4.

Atoms	(a)	(b)	(c)	(d)	(e)	*Hu et al.*[1]
C1	0.020	0.172	0.068	0.215	0.192	-0.100
H2	0.086	0.010	0.029	0.004	0.016	0.100
C3	-0.089	0.137	0.186	0.134	0.147	-0.100
H4	0.131	0.020	0.034	0.081	0.094	0.100
N5	-0.320	-0.567	-0.566	-0.596	-0.491	-0.500
C6	0.501	0.601	0.577	1.070	0.952	0.500
C7	-0.389			-0.694	-0.562	-0.300
H8	0.119	-0.048*	-0.061*	0.242	0.089	0.100
H9	0.124			0.112	0.225	0.100
H10	0.114			0.099	0.089	0.100
N11	-0.472	-1.131	-0.960	-1.358	-1.231	-0.500
Zn12	1.036	1.614	1.385	1.692	1.480	1.000
H13**	0.327	-	-	-	-	-

* The -CH_3 groups were replaced by *H* atoms to reduce the computational cost, since they pose little effect on the charges.
** H13 is bonded with N5 (NH group).

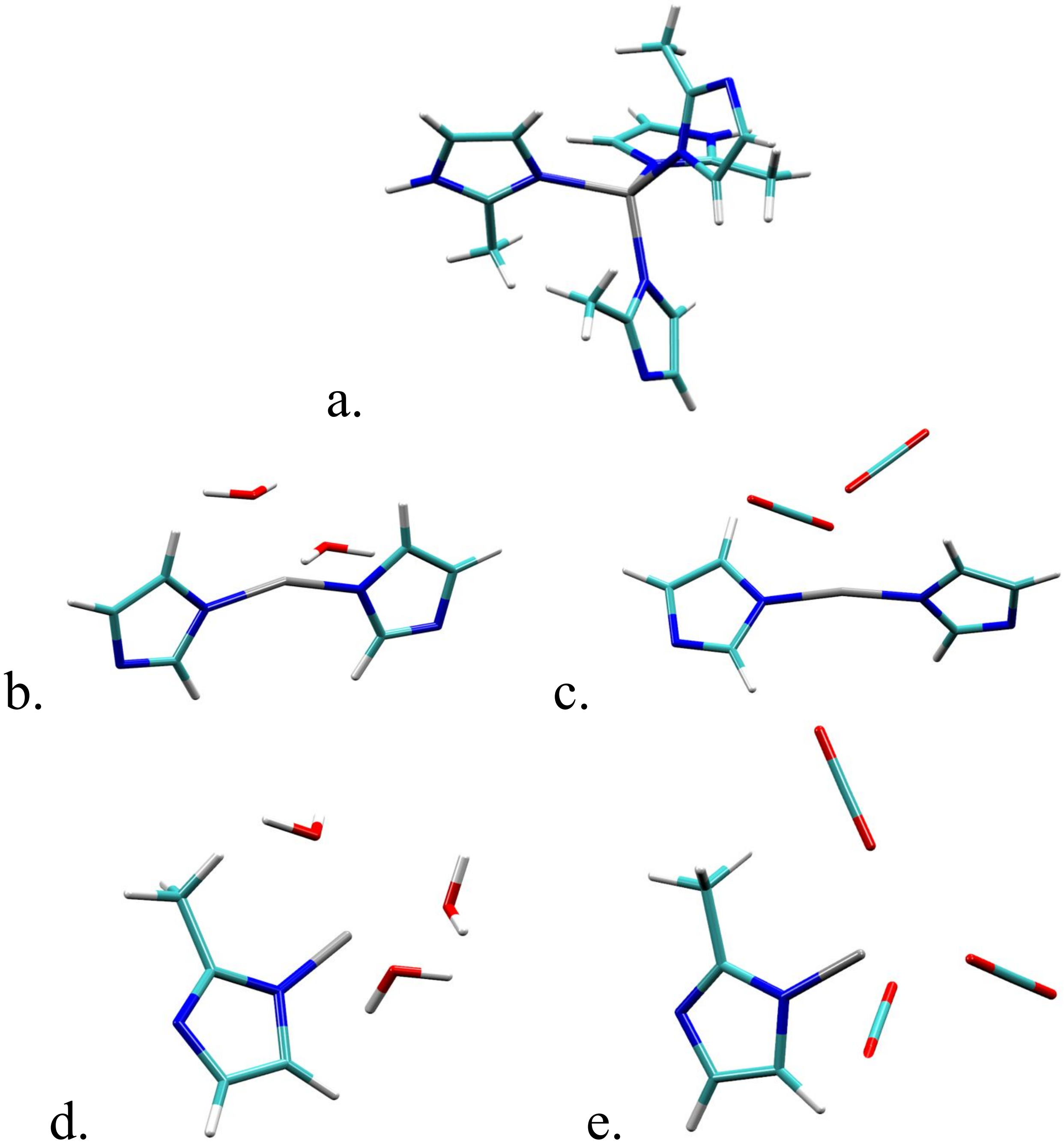

Figure S4: Optimized geometries obtained with B3LYP/aug-cc-pVDZ. Systems(a) $[Zn^{+2}(mIm^{-})_4 (H^{+})_2]$; (b) $[Zn^{+2}(Im^{-})_2]+(H_2O)_2$; (c) $[Zn^{+2}(Im^{-})_2]+(CO_2)_2$; (d) $[Zn^{+2}(mIm^{-})]+(H_2O)_3$; and (e) $[Zn^{+2}(mIm^{-})]+(CO_2)_3$. Clusters used in the atomic charge calculations.

S3. Optimized [$Zn^{+2}mIm^{-}$] clusters with CO_2, H_2O and N_2

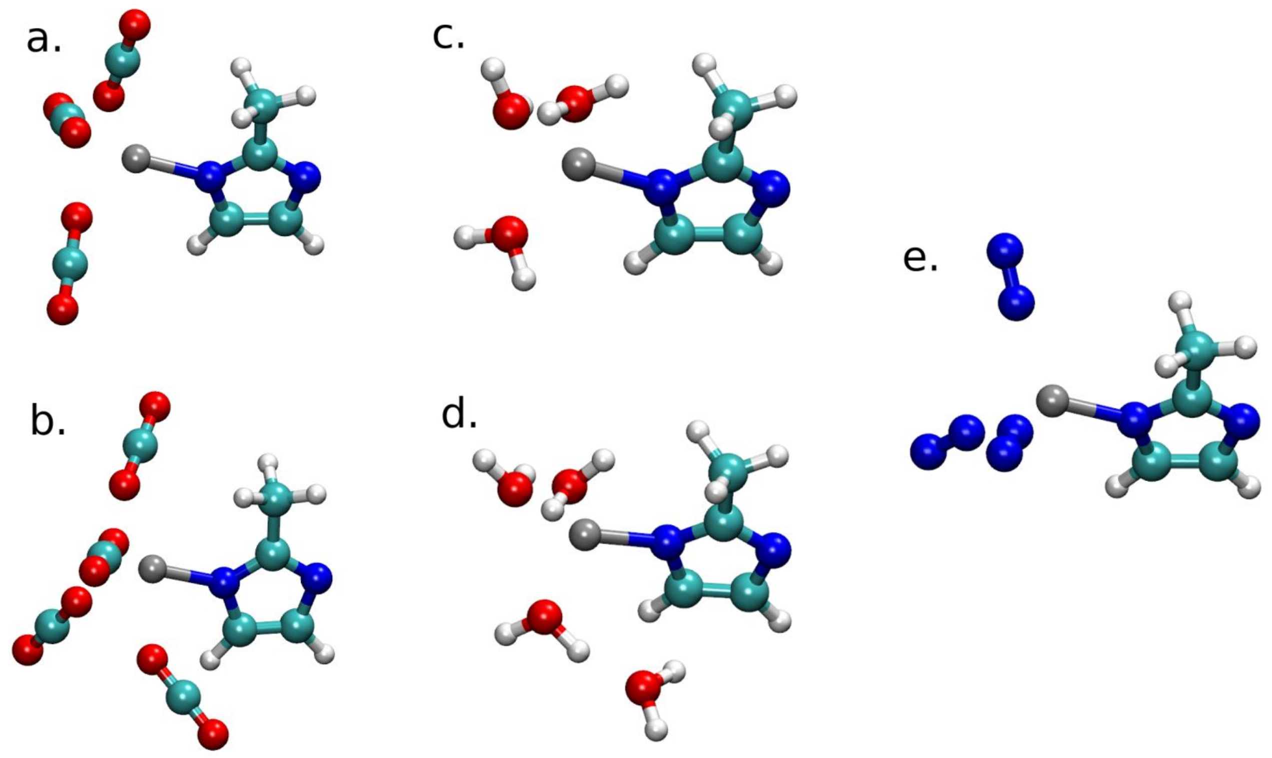

Figure S5: Optimized cluster with B3LYP/ aug-cc-pVDZ of [$Zn^{+2}mIm^{-}$] interacting with CO_2 molecules using $N_{gas} = 3$ (a) and $N_{gas} = 4$ (b); with H_2O molecules using $N_{gas} = 3$ (c) and $N_{gas} = 4$ (d); and with N_2 molecules using $N_{gas} = 3$ (e). Clusters used in the calculations of average binding energy $\langle E_{ij} \rangle$ and average distances between the Zn atom and the O atoms of the CO_2 and H_2O molecules, $\langle R_{Zn\text{-}O} \rangle$, and N atoms N_2, $\langle R_{Zn\text{-}N} \rangle$.

S4. Simulated systems

Table S3: Systems that were simulated with Monte Carlo method in this work.

Solvent	Gas	Ensemble	P (atm)	T (K)
$[(Zn^{+2})_{24} (mIm^{-})_{60} (Zn^{+2})_{24}]$	100 CO_2	NVT	1	273
$[(Zn^{+2})_{24} (mIm^{-})_{60} (Zn^{+2})_{24}]$	200 CO_2	NVT	1	273
$[(Zn^{+2})_{24} (mIm^{-})_{60} (Zn^{+2})_{24}]$	500 CO_2	NVT	1	273
$[(Zn^{+2})_{24} (mIm^{-})_{60} (Zn^{+2})_{24}]$	1000 CO_2	NVT	1	273
$[(Zn^{+2})_{24} (mIm^{-})_{60} (Zn^{+2})_{24}]$	2000 CO_2	NVT	1	273
$[(Zn^{+2})_{24} (mIm^{-})_{60} (Zn^{+2})_{24}]$	1000 CO_2	NVT	1	298
$[(Zn^{+2})_{24} (mIm^{-})_{60} (Zn^{+2})_{24}]$	1000 CO_2	NPT	1	273

$[(Zn^{+2})_{24}\ (mIm^{-})_{60}\ (Zn^{+2})_{24}]$	1000 CO_2	NPT	20	273
$[(Zn^{+2})_{24}\ (mIm^{-})_{60}\ (Zn^{+2})_{24}]$	1000 H_2O	NVT	1	273
$[(Zn^{+2})_{24}\ (mIm^{-})_{60}\ (Zn^{+2})_{24}]$	1000 H_2O	NVT	1	298
$[(Zn^{+2})_{24}\ (mIm^{-})_{60}\ (Zn^{+2})_{24}]$	1000 N_2	NVT	1	273
$[(Zn^{+2})_{24}\ (mIm^{-})_{60}\ (Zn^{+2})_{24}]$	1000 O_2	NVT	1	273
$[(Zn^{+2})_{24}\ (mIm^{-})_{60}\ (Zn^{+2})_{24}]$	1000 Ar	NVT	1	273
$[(Zn^{+2})_{24}\ (mIm^{-})_{60}\ (Zn^{+2})_{6}]$	1000 CO_2	NVT	1	273
$[(Zn^{+2})_{24}\ (mIm^{-})_{60}\ (Zn^{+2})_{6}]$	1000 H_2O	NVT	1	273
$[(Zn^{+2})_{24}\ (mIm^{-})_{60}\ (Zn^{+2})_{6}]$	1000 N_2	NVT	1	273
$[(Zn^{+2})_{24}\ (mIm^{-})_{60}\ (Zn^{+2})_{12}]$	1000 CO_2	NVT	1	273
$[(Zn^{+2})_{24}\ (mIm^{-})_{60}\ (Zn^{+2})_{12}]$	1000 H_2O	NVT	1	273
$[(Zn^{+2})_{24}\ (mIm^{-})_{60}\ (Zn^{+2})_{12}]$	1000 N_2	NVT	1	273
$[(Zn^{+2})_{24}\ (mIm^{-})_{60}\ (Zn^{+2})_{16}]$	1000 CO_2	NVT	1	273
$[(Zn^{+2})_{24}\ (mIm^{-})_{60}\ (Zn^{+2})_{16}]$	1000 H_2O	NVT	1	273
$[(Zn^{+2})_{24}\ (mIm^{-})_{60}\ (Zn^{+2})_{16}]$	1000 N_2	NVT	1	273
$[(Zn^{+2})_{24}\ (mIm^{-})_{60}\ (Zn^{+2})_{20}]$	1000 CO_2	NVT	1	273
$[(Zn^{+2})_{24}\ (mIm^{-})_{60}\ (Zn^{+2})_{20}]$	1000 H_2O	NVT	1	273
$[(Zn^{+2})_{24}\ (mIm^{-})_{60}\ (Zn^{+2})_{20}]$	1000 N_2	NVT	1	273
$[(Zn^{+2})_{24}\ (mIm^{-})_{60}\ (H^{+})_{12}]$	1000 CO_2	NVT	1	273
$[(Zn^{+2})_{24}\ (mIm^{-})_{60}\ (H^{+})_{12}]$	1000 H_2O	NVT	1	273
$[(Zn^{+2})_{24}\ (mIm^{-})_{60}\ (H^{+})_{12}]$	1000 N_2	NVT	1	273
$[(Zn^{+2})_{24}\ (mIm^{-})_{60}\ (H^{+})_{18}]$	1000 CO_2	NVT	1	273
$[(Zn^{+2})_{24}\ (mIm^{-})_{60}\ (H^{+})_{18}]$	1000 H_2O	NVT	1	273
$[(Zn^{+2})_{24}\ (mIm^{-})_{60}\ (H^{+})_{18}]$	1000 N_2	NVT	1	273
$[(Zn^{+2})_{24}\ (mIm^{-})_{60}\ (H^{+})_{24}]$	1000 CO_2	NVT	1	273
$[(Zn^{+2}]_{24}\ (mIm^{-}]_{60}\ (H^{+}]_{24})$	1000 H_2O	NVT	1	273
$[(Zn^{+2})_{24}\ (mIm^{-})_{60}\ (H^{+})_{24}]$	1000 N_2	NVT	1	273
$[Zn^{+2}\ mIm^{-}]$	100 CO_2	NVT	1	273
$[Zn^{+2}\ mIm^{-}]$	100 H_2O	NVT	1	273
$[Zn^{+2}\ mIm^{-}]$	100 N_2	NVT	1	273
-	2000 CO_2	NVT	1	273

-	2000 CO_2	NPT	1	273
-	2000 CO_2	NVT	10	225
-	2000 CO_2	NPT	10	225
-	2000 CO_2	NVT	72	298
-	2000 CO_2	NPT	72	298
$[(Zn^{+2})_{24} (mIm^{-})_{60} (Zn^{+2})_{24}]$	1000 CO_2 + 1000 H_2O	NVT	1	273
$[(Zn^{+2})_{24} (mIm^{-})_{60} (Zn^{+2})_{24}]$	1000 CO_2 + 1000 N_2	NVT	1	273

Table S4: Systems that were simulated with Molecular Dynamics method in this work.

Solvent	Gas	Ensemble	P (atm)	T (K)
$[(Zn^{+2})_{24} (mIm^{-})_{60} (Zn^{+2})_{24}]$	1000 N_2	NVT	1	273
$[(Zn^{+2})_{24} (mIm^{-})_{60} (Zn^{+2})_{24}]$	1000 CO_2	NVT	1	273
$[(Zn^{+2})_{24} (mIm^{-})_{60} (Zn^{+2})_{24}]$	1000 CO_2 + 1000 N_2	NVT	1	273
$[(Zn^{+2})_{24} (mIm^{-})_{60} (Zn^{+2})_{24}]$	1000 CO_2 + 9000 N_2	NVT	1	273
$[(Zn^{+2})_{144} (mIm^{-})_{336} (Zn^{+2})_{72}]$	8000 CO_2	NVT	1	273
$[(Zn^{+2})_{144} (mIm^{-})_{336} (Zn^{+2})_{24}]$	8000 CO_2	NVT	1	273
$[(Zn^{+2})_{432} (mIm^{-})_{972} (Zn^{+2})_{144}]$	27000 CO_2	NVT	1	273

Table S5: Systems that were simulated with Born Oppenheimer Molecular Dynamics method in this work.

Solvent	Gas	T (K)
$[Zn^{+2}\ mIm^{-}]$	4 CO_2	298
$[Zn^{+2}\ mIm^{-}]$	4 H_2O	298

S5. ZIF-8 flexibility

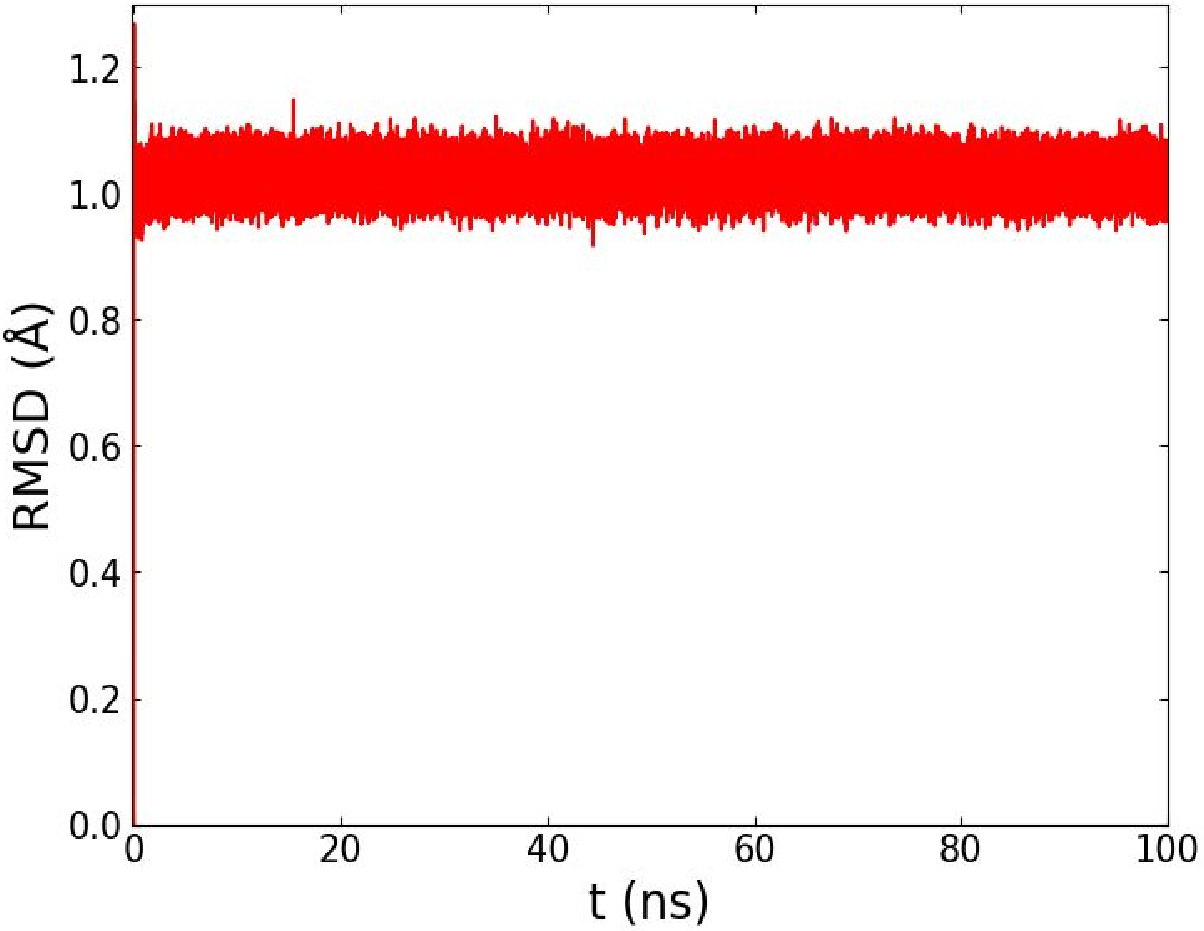

Figure S6: RMSD of the flexible 1x1x1 nanoparticle of ZIF-8 simulated by Molecular Dynamics using the leap-frog integrator with 2 fs of time step, the LINCS algorithm for the Hydrogen bonds constraints, the velocity rescaling thermostat with coupling constant of 0.1 ps, the cut-off radius of 6 nm, the NVT ensemble with temperature of 273 K and pressure of 1 bar, and total time simulation of 100 ns.

S6. Solvation of the ZIF-8 nanoparticle

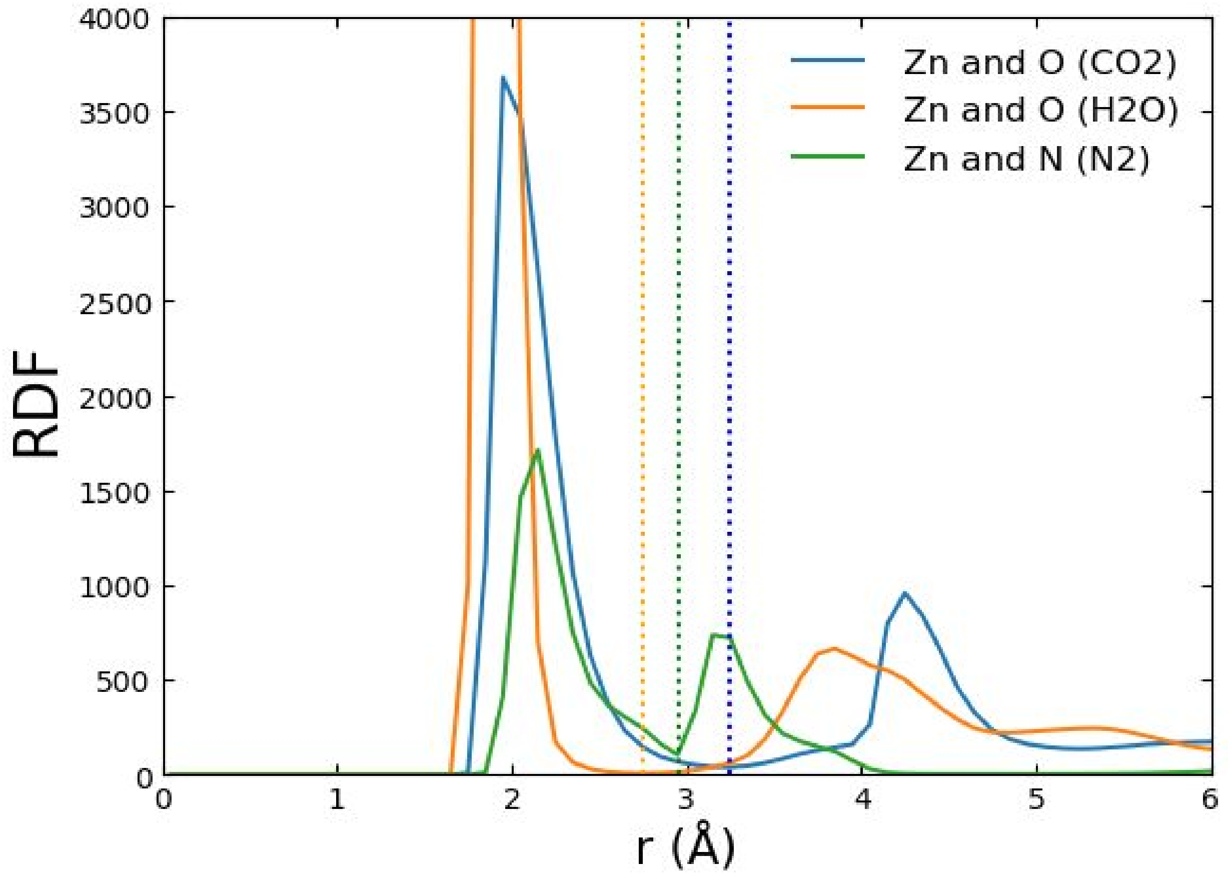

Figure S7: Radial distribution function obtained from MC simulations of 1x1x1 ZIF-8 nanoparticle + 1000 gas molecules: N_2 in green, CO_2 in blue and H_2O in orange. These distributions are for the Zn atoms on the surface sites and Nitrogen from N_2 gas and Oxygen from CO_2 and H_2O gases. The vertical dashed lines defined the first solvation shell of the ZIF-8 nanoparticle: 2.75 Å for H_2O, 2.95 Å for N_2 and 3.25 Å for CO_2.

References

[1] Hu, Z.; Zhang, L.; Jiang, J. Development of a force field for zeolitic imidazolate framework-8 with structural flexibility, *J. Chem. Phys.*, **2012**, *136*, 244703.

[2] Cezar, H. M.; Canuto, S.; Coutinho, K. DICE: A Monte Carlo Code for Molecular Simulation Including the Configurational Bias Monte Carlo Method, *J. Chem. Inf. Model.*, **2020**, *60*, 3472-3488.
